# Glucose Deprivation‐Induced Disulfidptosis via the *SLC7A11‐INF2* Axis: Pan‐Cancer Prognostic Exploration and Therapeutic Validation

**DOI:** 10.1002/advs.202408556

**Published:** 2025-07-25

**Authors:** Zhenyu Song, Qiuming Yao, Lina Huang, Dan Cui, Jun Xie, Leilei Wu, Jianfeng Huang, Bo Zhai, Dan Liu, Xiao Xu

**Affiliations:** ^1^ Mini‐invasive Interventional Therapy Center Shanghai East Hospital Tongji University 200120 Shanghai China; ^2^ GMU‐GIBH Joint School of Life Sciences The Guangdong‐Hong Kong‐Macao Joint Laboratory for Cell Fate Regulation and Diseases The Affiliated Traditional Chinese Medicine Hospital Guangzhou Medical University Guangzhou Guangdong Province 511436 China; ^3^ Department of Obstetrics and Gynecology Zhongshan Hospital Fudan University Shanghai 200032 China; ^4^ Department of General Practice Zhongshan Hospital Fudan University Shanghai 200032 China; ^5^ Shanghai Key Laboratory of Metabolic Remodeling and Health Institute of Metabolism and Integrative Biology Fudan University Shanghai 200032 China; ^6^ Department of Rehabilitation Medicine The Affiliated Hospital of Youjiang Medical University for Nationalities Baise 533000 China; ^7^ Key Laboratory of Research and Development on Clinical Molecular Diagnosis for High‐Incidence Diseases of Baise Baise Guangxi 533000 China; ^8^ Department of Radiation Oncology Shanghai Pulmonary Hospital School of Medicine Tongji University Shanghai 200433 China; ^9^ Department of Radiology Zhongshan Hospital, Fudan University Shanghai 200032 China

**Keywords:** disulfidptosis, INF2, ovarian cancer, pan‐cancer analysis, prognosis, SLC7A11

## Abstract

Disulfidptosis, a novel form of regulated cell death, involves cytoskeletal collapse due to excessive disulfide bond formation, linking metabolism and reactive oxygen species to potential cancer therapy targets. Recent multi‐omics studies highlight the prognostic value of disulfidptosis‐related gene (DRG) signatures in pan‐cancers; however, the molecular mechanisms underlying their biological functions and therapeutic relevance remain poorly defined. Herein, a DRG score model is constructed using LASSO Cox regression across 33 cancer types, and a nomogram incorporating the DRG score is developed for prognostic prediction. The tumor microenvironment, mutation profiles, and immunotherapy responses are analyzed. The DRG score serves as an independent prognostic factor across cancers, correlating with poor outcomes and malignant features. Glucose deprivation induces disulfidptosis in *SLC7A11* ^high^ cells (high *SLC7A11* expression), especially in cancers with a high DRG score, such as ovarian cancer. Silencing *INF2* prevents disulfidptosis and decreases susceptibility to irofulven, which can be reversed by GLUT inhibitors. *SLC7A11* knockdown reduces disulfidptosis, restores ATP/NADPH levels, and protects the cytoskeleton under glucose deprivation, whereas *INF2* knockdown impairs cell migration. Moreover, the DRG scores predict prognosis and therapeutic responses. The *SLC7A11‐INF2* axis regulates disulfidptosis, migration, and drug sensitivity, highlighting its potential as a marker of metabolic vulnerability in ovarian cancer.

## Graphical abstract

Glucose deprivation or GLUT1 inhibition induces disulfidptosis in *SLC7A11* ^high^ ovarian cancer cells by promoting cystine accumulation, NADPH/ATP depletion, and F‐actin disulfide formation. SLC7A11 interacts with INF2 to further increase H₂O₂ levels and impair mitochondrial fission, suppressing cell migration. Targeting the *SLC7A11–INF2* axis represents a promising redox‐based therapeutic strategy for aggressive ovarian cancer.

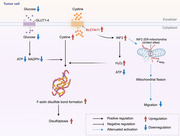

## 1 Introduction

Cancer remains a leading cause of death worldwide, with over 2 million new cases and more than 600 000 deaths in the United States in 2024.**^[^** ^1^, ^2^, ^3^ **^]^** While therapeutic advances have improved the survival of patients with some cancers, many aggressive malignancies, including ovarian cancer (OV), continue to show poor long‐term outcomes. As a typical example of a treatment‐resistant gynecological tumor, OV remains a major clinical concern. It ranks sixth in cancer‐related mortality among women in the US and eighth globally, with a 5‐year survival rate of only ≈30% for advanced‐stage disease.^[^ ^4^ ^]^ Standard OV treatment consists of cytoreductive surgery followed by platinum‐based chemotherapy,^[^ ^5^ ^]^ with maintenance therapy using PARP inhibitors such as niraparib, providing progression‐free survival benefits regardless of homologous recombination status.^[^ ^6^ ^]^ However, most patients eventually relapse, and therapeutic resistance remains a major obstacle. These limitations underscore the urgent need for more durable and tumor‐specific treatment strategies for OV. Therapeutic resistance is increasingly linked to factors beyond tumor cells, including the tumor microenvironment (TME).^[^ ^7^ ^]^ Looking ahead, future cancer therapies will increasingly rely on integrating molecular profiling and metabolic targeting to address context‐specific vulnerabilities.^[^ ^8^ ^]^

Against this therapeutic backdrop, “disulfidptosis,” a novel form of regulated cell death, was first described in 2023 to involve disulfide stress‐induced cytoskeletal collapse under glucose deprivation.^[^ ^9^ ^]^ Disulfidptosis is governed by a set of molecular pathways and is susceptible to genetic and pharmacological modulation.^[^ ^10^ ^]^ Glucose deprivation in tumor cells with high expression of solute carrier family 7 member 11 (SLC7A11), a cystine transporter, can trigger disulfidptosis.^[^ ^1^ ^]^ When cells are deprived of glucose, elevated SLC7A11 activity may deplete nicotinamide adenine dinucleotide phosphate (NADPH) levels and induce disulfide stress. This stress promotes the establishment of disulfide bonds among the sulfhydryl groups of cystine residues in certain proteins, impairing their regular activities and functions, and ultimately driving toward cell death.^[^ ^9^ ^]^ Beyond its role in metabolic vulnerability, disulfidptosis may also contribute to immune escape and chemoresistance.^[^ ^11^ ^]^ When CD8+ T cells undergo disulfidptosis, their exhaustion impairs antitumor immunicty.^[^ ^12^ ^]^ Moreover, disruption of actin cytoskeleton integrity can impair immune synapse formation and reduce T cell activation and tumor clearance.^[^ ^13^ ^]^ In parallel, antioxidant buffering driven by *SLC7A11* overexpression may suppress reactive oxygen species (ROS)‐mediated cytotoxicity, diminishing the efficacy of many chemotherapeutic agents.^[^ ^13^, ^14^ ^]^ Furthermore, this understanding opens up potential avenues for novel interventions in metabolic cancer therapy.

SLC7A11 enhances glutathione synthesis and reduces oxidative stress under nutrient‐replete conditions. The *SLC7A11* gene suppresses ferroptosis and is upregulated in various types of cancers.^[^ ^15^ ^]^ In 2017, it was reported that SLC7A11 dramatically sensitized cell death in the absence of glucose.^[^ ^16^ ^]^ Considering that the WAVE regulatory complex only mildly hinders disulfidptosis, disturbances in disulfide bonds could influence diverse proteins, such as those constituting the actin cytoskeleton.^[^ ^2^ ^]^ Additionally, Yan et al. highlighted that high‐level overexpression of *SLC7A11* sensitizes cancer cells to hydrogen peroxide‐induced cell death, compared with moderate overexpression, like through disulfidptosis.^[^ ^14^ ^]^ The impact of *SLC7A11* on cell fate is shaped by both its expression level and the metabolic context. For instance, inhibitors of the glucose transporter (GLUT) effectively diminish the cellular uptake of glucose, leading to heightened disulfide cross‐linking in proteins associated with the actin cytoskeleton, skeletal muscle contraction, and the induction of disulfidptosis in cancer cells exhibiting elevated *SLC7A11* expression. Disulfidptosis predominantly occurs in these cells because of the inadequate generation of NADPH necessary for converting cystine into cystine, which sparks disulfide stress.^[^ ^17^ ^]^ There is mounting evidence indicating that triggering disulfidptosis could serve as a potent therapeutic strategy for treating various cancers. Therefore, it is crucial to explore the role of disulfidptosis in cancer development and its response to different therapeutic interventions.

Recent investigations have revealed that disulfidptosis‐related genes (DRG) play a role in various cancers, such as liver hepatocellular carcinoma (LIHC), bladder urothelial carcinoma (BLCA), breast invasive carcinoma (BRCA), and lung adenocarcinoma (LUAD).^[^ ^18^, ^19^, ^20^, ^21^ ^]^ However, the specific molecular mechanisms underlying these processes and their therapeutic implications remain unclear. To address this gap, we performed a comprehensive pan‐cancer analysis of DRGs using transcriptomic data from primary tumors. By applying a Least Absolute Shrinkage and Selection Operator (LASSO) Cox regression model, we constructed a robust disulfidptosis‐related gene signature (DRGS) with broad applicability across multiple malignancies. A nomogram incorporating the DRG scores as a quantitative tool was also developed to predict patient prognosis. A robust link was observed between elevated DRG scores and poor clinical outcomes across various types of cancers. OV cells with high DRG scores predominantly comprise *SLC7A11* ^high^ cells. In vitro studies on the SKOV3 OV cell line revealed that cells with high *SLC7A11* expression underwent disulfidptosis under glucose‐deprived conditions. DRG score analysis revealed the marked upregulation of the inverted formin 2 (*INF2*) gene across various female gonadal tumors, especially in OV cell lines. *INF2*, which encodes a member of the formin family of proteins, plays a crucial role in regulating actin filament dynamics regulation through its inhibitory domain.^[^ ^22^, ^23^ ^]^ Increased *INF2* expression has been associated with mitochondrial fission in endometrial, prostate, and thyroid cancers.^[^ ^24^, ^25^, ^26^ ^]^ In this study, functional experiments further demonstrated that *INF2* participated in the disulfidptosis and modulates the responsiveness of OV cells to treatment. Our study enhances the understanding of the role of the DRGS in a broad array of cancers and underscores the potential of the *SLC7A11‐INF2* axis as biomarkers and therapeutic targets, particularly in metabolically vulnerable tumors such as OV.

## 2 Result

### 2.1 A High Disulfidptosis Score Indicates a Poor Clinical Prognosis

**Figure 1:**
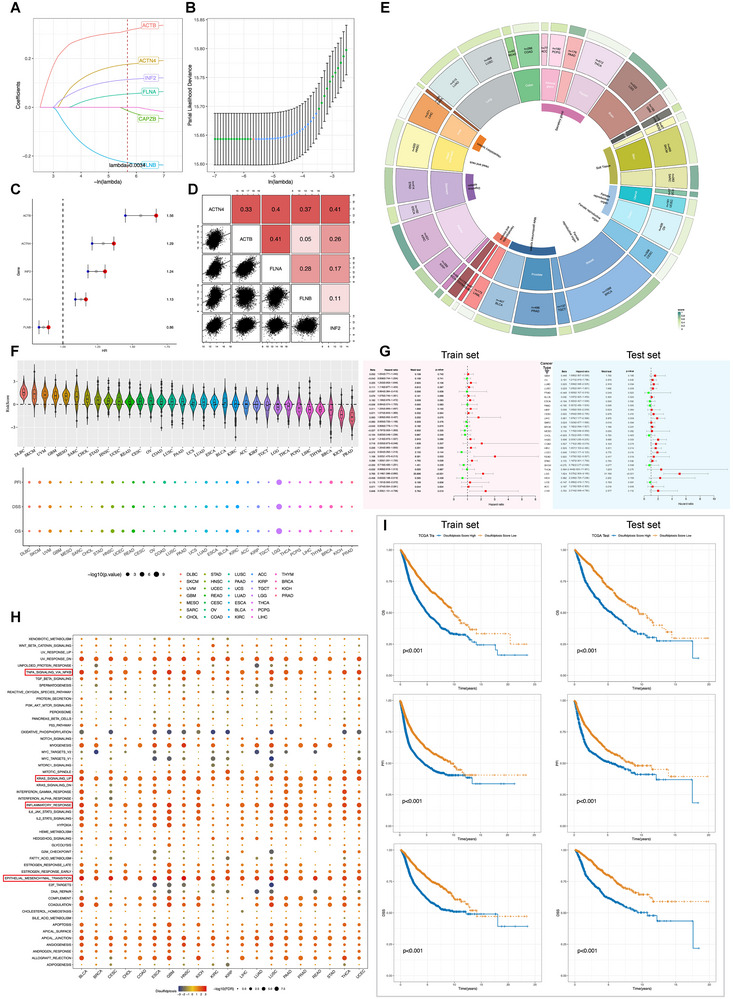
Construction and prognostic performance evaluation of the disulfidptosis‐reglated gene signature (DRGS) for pan‐cancer. A) DRG‐initial biomarkers were put into the least absolute shrinkage and selection operator (LASSO) regression analysis in the Cancer Genome Atlas (TCGA) pan‐cancer training set. B) Cross‐validation of the constructed signature. C) The hazard ratio of every single gene for the prognosis was observed. D) Pair‐wise correlation analysis was performed on these genes. E) The TCGA database systematically provided risk levels for 33 types of cancer. F) Panorama of DRG‐risk score in pan‐cancers. G) The training and testing cohorts employed univariate Cox analyses to assess the impact of DRG score on different predictive prognoses. H) The Gene Set Enrichment Analysis was utilized to elucidate the association between cancer‐related cellular signaling pathways and disulfidptosis. I) The disease‐specific survival (DSS), overall survival (OS), and progression‐free interval (PFI) were evaluated in both the TCGA training and testing sets.

The Cancer Genome Atlas (TCGA) dataset was divided into training and validation sets to evaluate the prognostic value of the genes via univariate Cox regression analysis. Subsequently, LASSO regression was applied to refine the list to six notable genes with a significant lambda value of 0.0034, which was selected using the one‐standard‐error rule to balance model simplicity and performance (**Figure** **1**A). A stepwise selection process was employed in the subsequent multivariate Cox regression analysis (Figure 1B). Finally, five genes were selected to establish a predictive model. The risk score equation of the model was as follows: Risk Score = 0.191 × *ACTN4* + 0.316 × *ACTB* + 0.062 × *FLNA* – 0.246 × *FLNB* + 0.124 × *INF2*. Following the standardization of the risk scores, the samples were classified into high‐ or low‐risk groups. The five‐gene DRG score derived from Cox regression was correlated with survival outcomes (Figure 1C). Correlation analyses were then conducted on the five DRGs (Figure 1D). A comprehensive evaluation of the 33 cancer types in the TCGA data revealed organ‐specific risk profiles displayed in concentric circles, varying from the innermost to the outermost, representing tissue and cancer types and their corresponding risk scores (Figure 1E). The DRG risk score presented specific patterns; for instance, higher scores were associated with ovarian and uterine cancers, whereas lower scores were associated with liver, prostate (PRAD), thyroid (THCA), and breast (BRCA) cancers (Figure 1F). Interestingly, regarding patient outcomes, the DRG scores varied within the same tissue type; higher scores indicated poorer prognosis, specifically in uterine corpus endometrial carcinoma (UCEC), but not in cervical squamous cell carcinoma and endocervical adenocarcinoma (CESC), or uterine carcinosarcoma (UCS). Patients with UCEC regularly showed elevated DRG scores, implying that disulfidptosis may affect tumor behavior differently depending on the organ type and subtype. The prognostic significance of the DRG scores in various cancers was confirmed using univariate Cox analysis in both the training and validation sets (Figure 1G). Furthermore, geneset enrichment analysis (GSEA) revealed that tumors with high disulfidptosis scores were significantly enriched in pathways associated with epithelial–mesenchymal transition (EMT), KRAS signaling, TNF‐α signaling, and inflammatory response (Figure 1H). Consistent with our findings, recent studies have also reported the occurrence of disulfidptosis in inflammatory contexts, such as psoriasis,^[^ ^27^ ^]^ and in stromal components like cancer‐associated fibroblasts.^[^ ^28^ ^]^ These external observations lend additional support to the inflammatory pathway enrichment identified in our high‐disulfidptosis score group, thereby reinforcing the biological relevance of our model. Notably, our analysis revealed a strong association between disulfidptosis and KRAS signaling, a link not previously reported in other pan‐cancer disulfidptosis studies. This finding reflects the broader sensitivity of our model for detecting significant pathways. Given the growing application of KRAS‐targeted therapies, such as AMG510 and MRTX849, in tumors,^[^ ^29^ ^]^ our results suggest that disulfidptosis may have potential clinical relevance as a targetable metabolic vulnerability. Based on their median DRG scores, patients were grouped into the high‐ and low‐risk groups; those with high scores typically exhibited worse survival metrics, including the disease‐specific survival (DSS), overall survival (OS), and progression‐free interval (PFI) (Figure 1I); The corresponding Kaplan‐Meier curves from the training set are provided in Figure S1 (Supporting Information). Overall, the DRG score is a robust and broadly applicable prognostic marker across various cancer types.

### 2.2 Development of a Prognostic Nomogram Incorporating the DRG Score for Pan‐Cancer Survival Prediction and Determination of Malignant Features

**Figure 2:**
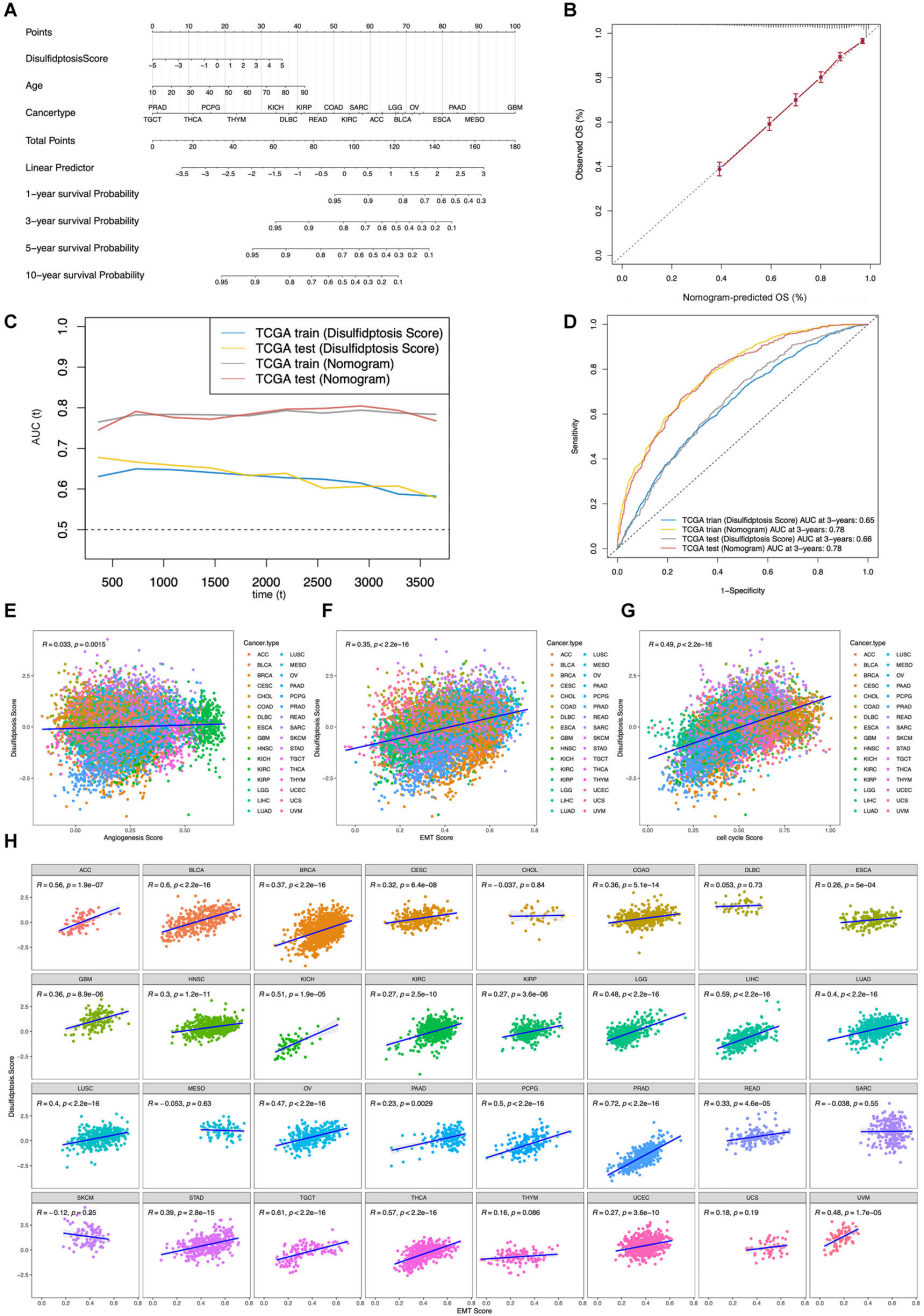
Enhanced evaluation of the DRG‐risk score and its derived nomogram signature for predicting pan‐cancer prognosis and malignant tumor features. A) The nomogram integrating the DRG‐risk score and clinicopathological characteristics. B) The calibration curve of the 3‐ and 5‐year OS. C, D) The area under the curve (AUC) of the nomogram and DRGS prediction was evaluated in both the training and the testing cohorts. The correlation analyses were performed between the E) DRG score and angiogenesis z‐score, F) cell cycle z‐score, G) and EMT z‐score in the overall TCGA pan‐cancer cohort. H) The correlation between the DRG score and EMT score in individual cancer types.

To quantify the DRG scores for the clinical prediction of adverse outcomes, we devised a detailed nomogram that integrated the DRG score with various clinicopathological parameters, such as patient age and specific cancer diagnoses (**Figure** **2**A). The prognostic accuracy of our model was suggested by the calibration curves for 3‐ and 5‐year OS, which closely matched the ideal curve depicted by a unity‐sloped line intersecting the origin, as shown in Figure 2B. These results demonstrated a commendable correspondence between the predicted and observed survival outcomes. Comparative analysis of the area under the receiver operating characteristic (ROC) curves for the survival predictions in both training and validation cohorts revealed the superior performance of the nomogram containing multiple clinical factors compared to the DRG score alone (TCGA_train: 0.78 versus 0.65; TCGA_test: 0.78 versus 0.66), confirming that the multifactorial model enhances prognostic precision (Figures 2C,D). The paradigm of malignant transformation, often characterized by accelerated cellular growth, potentiated EMT, and augmented angiogenic capabilities, encapsulates critical oncogenic features. To examine the association between disulfidptosis and these cancer hallmarks, we employed a z‐score methodology to analyze datasets for angiogenesis, EMT, and cell cycle indices. Analysis across the TCGA pan‐cancer dataset revealed notable positive associations between disulfidptosis z‐scores and both cell cycle (R = 0.49, *p* < 0.05) and EMT progression (R = 0.354, *p* < 0.05), with a less pronounced linkage for angiogenesis (R = 0.033, *p* < 0.05) (Figures 2E‐G). The interplay between disulfidptosis and the EMT index across 33 different cancer types is depicted in Figure 2H, while Figure S2 (Supporting Information) provides insight into the correlations between cell cycle progression and angiogenesis. These findings underscore the association of tumor DRG scores with increased EMT activity and heightened malignant propensity.

### 2.3 *INF2* Exhibits a Strong Correlation with the Tumor Microenvironment, Immune Cell Infiltration, and Drug Sensitivity in Pan‐Cancers

**Figure 3:**
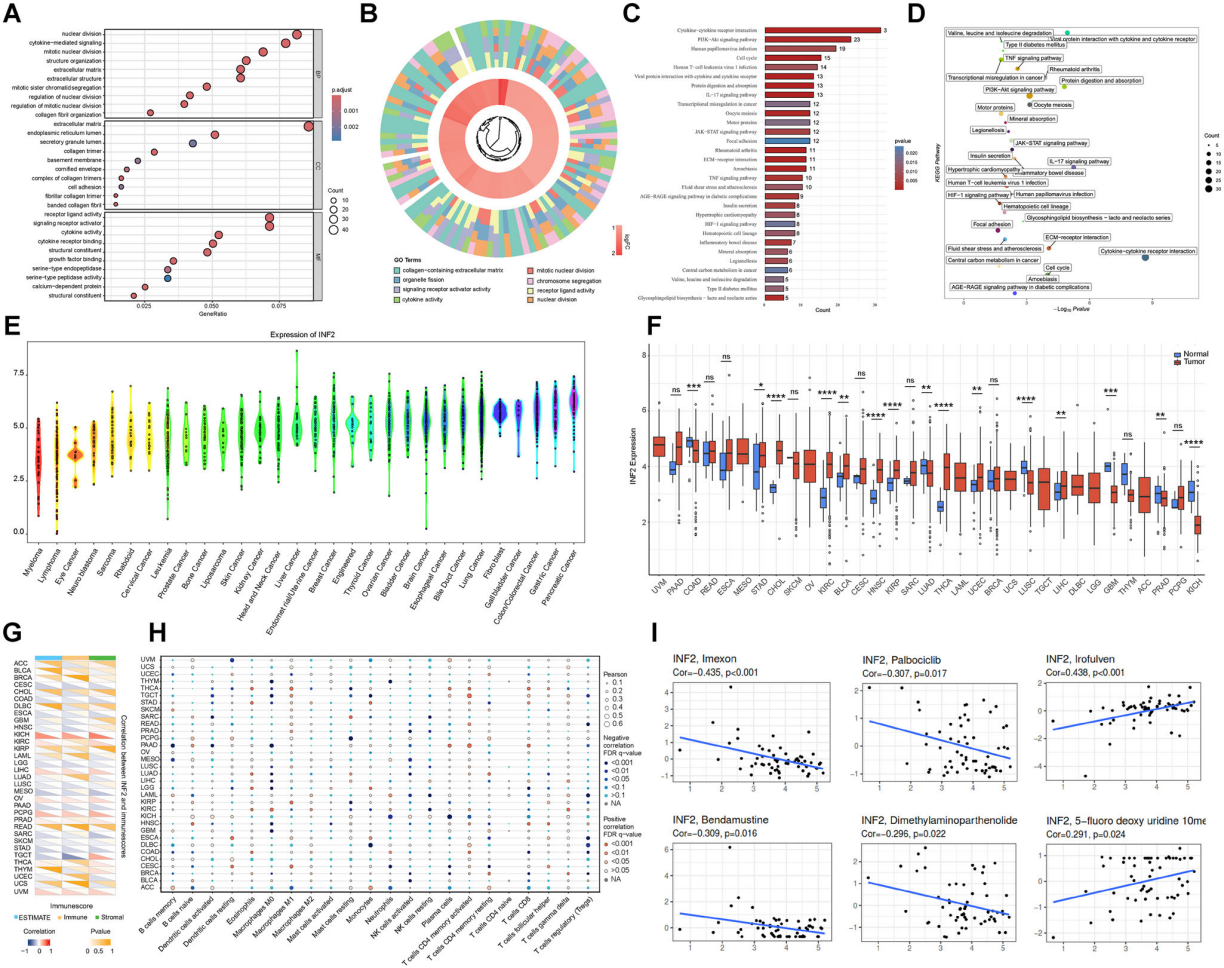
Correlation between INF2 expression, immune score, and drug sensitivity across 33 types of tumors. A,B) Gene Ontology (GO) analysis reveals significant and commonly enriched pathways in TCGA cohorts. C,D) Kyoto Encyclopedia of Genes and Genomes (KEGG) pathway analysis, visualized using bar plots and scatter plots, shows significant and commonly enriched pathways. E) The expression levels of INF2 were assessed across various tumor cell lines using the Cancer Cell Line Encyclopedia (CCLE) database from the Broad Institute. F) The expression levels of INF2 were analyzed in TCGA cohorts, comparing tumor and normal tissue across 33 different types of tumors. The relationship between INF2 expression levels and G) immune score, H) tumor immune infiltration, and I) drug sensitivity was investigated across 33 types of tumors. *p < 0.05; **p < 0.01; ***p < 0.001; ****p < 0.0001; ns, not significant.

To explore the relationship between tumor behavior and the immune landscape, patients were categorized into high‐risk (*n* = 4962) and low‐risk (*n* = 4302) groups based on their median DRG score. Differential expression genes (DEGs) analysis was performed with a cutoff of |log2FC| ≥ 1 and false discovery rate (FDR) < 0.05. Gene ontology (GO) enrichment analysis highlighted that these DEGs participated in cytokine receptor interactions, nuclear division, and extracellular matrix organization (**Figures** **3**A,B). Kyoto Encyclopedia of Genes and Genomes (KEGG) pathway enrichment further supported the involvement of these DEGs in cytokine signaling and the cell cycle (Figures 3C,D). Among the five DRGs, *INF2* emerged as a particularly noteworthy candidate. *INF2* encodes a member of the formin protein family and regulates actin polymerization^[^ ^30^ ^]^ F‐actin cytoskeletal collapse is a defining feature of disulfidptosis. Notably, Metascape analysis (https://metascape.org/) revealed that the biological processes enriched for *SLC7A11*—a known prerequisite for disulfidptosis—and *INF2* are mechanistically linked to metabolic and redox‐related pathways, suggesting a potential role for *INF2* in metabolic regulation and immune modulation during cancer progression (Table S1, Supporting Information). While several bioinformatics studies have implicated *INF2* as a potential candidate gene in disulfidptosis,^[^ ^31^, ^32^ ^]^ its direct biological role in this process has not yet been established. Therefore, we aimed to investigate whether *INF2* functionally contributes to disulfidptosis in cancer. Our DRG score‐based analysis revealed that *INF2* was notably upregulated in female gonadal tumors, particularly ovarian and endometrial/uterine cancers. Figure 3E shows the expression of *INF2* across various cancer cell lines derived from the Cancer Cell Line Encyclopedia (CCLE). Transcriptomic data from the TCGA dataset across 33 cancer types showed *INF2* is frequently upregulated in multiple malignancies (Figure 3F). In endometrial cancer, *INF2* has been shown to facilitate DRP1 recruitment to mitochondria, thereby promoting mitochondrial fission and enhancing tumor cell migration.^[^ ^31^ ^]^ However, its role in OV remains unexplored. We therefore conducted subsequent functional experiments to assess whether *INF2* contributes to disulfidptosis in OV cells. The other four DRGs, *ACTB*, *FLNA*, *FLNB*, and *ACTN4*, have previously been associated with tumorigenesis in liver,^[^ ^33^, ^34^, ^35^, ^36^, ^37^ ^]^ ovarian,^[^ ^38^, ^39^, ^40^, ^41^ ^]^ and endometrial cancers.^[^ ^42^, ^43^ ^]^ Using the ESTIMATE package in R, the immune and stromal components of the TME were examined. We observed a significant positive association between *INF2* levels and the ImmuneScore, ESTIMATEScore, and StromalScore in various cancer types (Figure 3G). The correlations between the remaining hub DRGs and immune infiltration levels are shown in Figures S3–S4 (Supporting Information). TME analysis in OV demonstrated a robust correlation between *INF2* expression and the DRG score, TME, and immune response markers (Figure S5, Supporting Information). Further investigation of the *INF2*‐linked immune cell infiltration showed positive correlations with M1 Macrophages, activated CD4+ memory T cells, CD8 T+ cells, and γδ T cells in most cancer types (Figure 3H). The relationship between *INF2* expression and response to anticancer drugs was also explored using CellMiner, where variations in *INF2* mRNA levels were linked to alterations in drug resistance or sensitivity (Figure 3I; Figure S6, Supporting Information). Notably, the enhanced expression of *INF2* was correlated with an increased response to irofulven (cor > 0.438, *p* < 0.05) and 5‐fluorodeoxyuridine 10‐mer, but decreased sensitivity to imexon, bendamustine, palbociclib, and dimethylaminoparthenolide. Gene co‐expression investigations recognized correlations between *INF2* expression and genes related to critical pathways like TNF‐β, TNF‐α signaling, hypoxia, pyroptosis, DNA repair, autophagy, and ferroptosis (Figure S7, Supporting Information). Associations between the remaining four DRGs and various tumor‐related pathways are documented in Figures S8–S11 (Supporting Information).

### 2.4 *INF2* is Associated with the Tumor Mutation Burden (TMB) in Cancer Rather Than the Efficacy of Immunotherapy

**Figure 4:**
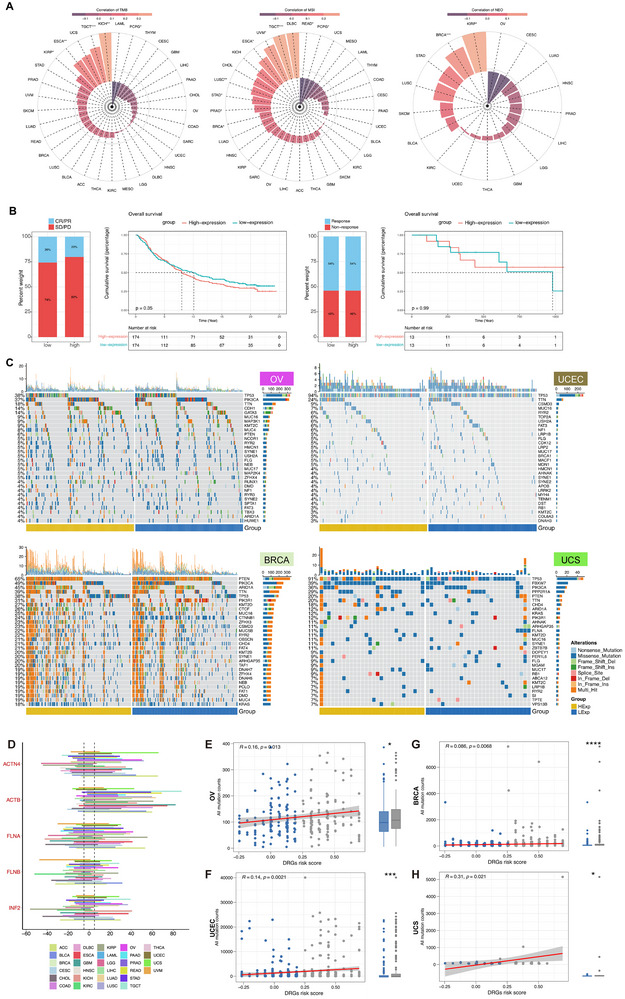
INF2 demonstrated significant associations with tumor mutational burden (TMB), microsatellite instability (MSI), neoantigen load (NEO), and tumor mutation status. Pan‐cancer expression, tumor mutational burden (TMB), microsatellite instability (MSI), A) neoantigens, B) immune checkpoint blockade therapy, C) as well as mutation profiles. D) The histogram illustrates the frequency of somatic copy number alterations for hub DRG in each cancer type. E–H) The association between cumulative mutation counts and the risk score of DRGs in female reproductive cancers. *p < 0.05; ***p < 0.001; ****p < 0.0001.

Given our findings so far, *INF2* stands out as a compelling candidate for further validation in functional experiments. The association between *INF2* mRNA levels and immunotherapy indicators such as TMB, microsatellite instability (MSI), and neoantigens (NEO) was investigated.^[^ ^44^ ^]^ A robust correlation was observed between the expression of *INF2* and these biomarkers across multiple cancer types (**Figure** **4**A), which was further supported by data from additional DRG hub genes (Figure S12, Supporting Information). Although not statistically significant, a trend toward poorer survival was observed in tumors with high *INF2* expression in the pan‐cancer analyses. This nonsignificant trend was also observed in the GSE78220 dataset (Figure 4B; Figure S13, Supporting Information), which may be partly attributed to the limited sample size and heterogeneity in tumor types. In ovarian, breast, and uterine cancers, elevated levels of *INF2* were associated with increased mutation rates in various mutation categories (Figure 4C; Figure S14, Supporting Information). Additionally, the relationship between disulfidptosis‐associated genomic instability and somatic copy number alterations (SCNAs) was studied, showing widespread occurrences in these cancers and a strong correlation with the mutation load, particularly in ovarian, breast, and uterine cancers (Figures 4D–H). Therefore, the association of *INF2* with genomic instability, immune modulation, and disulfidptosis‐related pathways provides a strong rationale for investigating its role in tumor migration, EMT regulation, and therapy resistance. Functional experiments should focus on elucidating the mechanistic involvement of *INF2* in disulfidptosis and its potential as a therapeutic target, particularly in cancers in which its expression is significantly altered.

### 2.5 *SLC7A11* ^high^ OV Cells Undergo Redox‐Dependent Disulfidptosis Induced By Glucose Starvation and GLUT Inhibition

**Figure 5:**
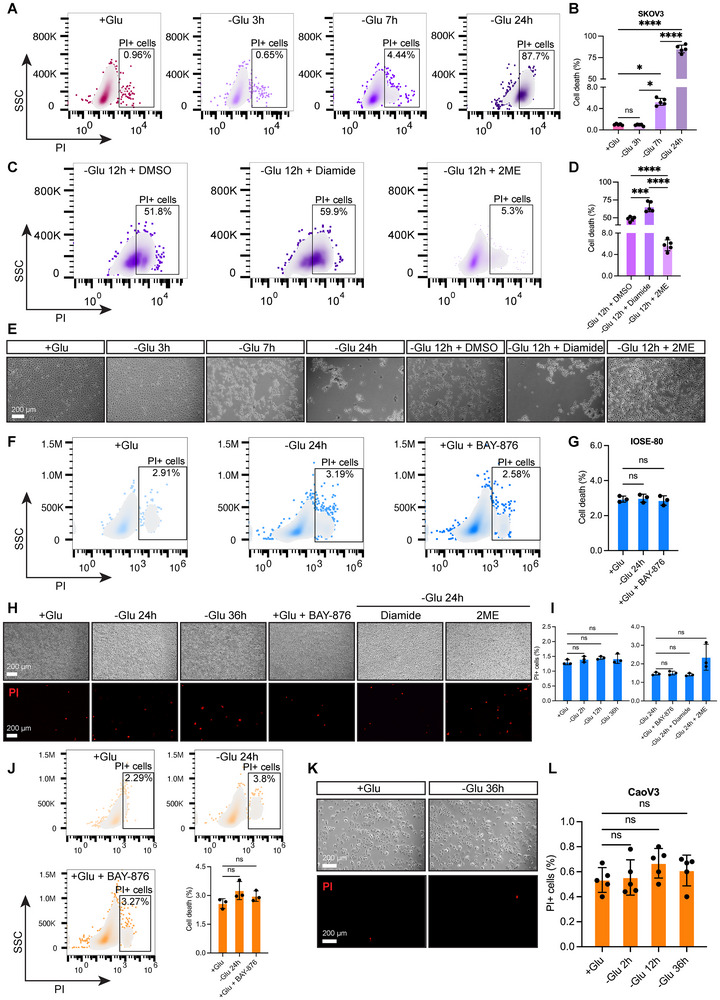
Effects of glucose deprivation on cell death in three different ovarian cancer cell lines. A) PI staining combined with flow cytometry analysis of the percentage of PI+ cells in SKOV3 cells at different time points of glucose deprivation, with representative flow cytometry density plots and corresponding B) quantitative analysis (*n* = 5 per group). C) After 12 h of glucose deprivation, the percentage of PI+ cells in SKOV3 cells was analyzed following treatment with Diamide (a disulfide bond accumulation inducer) and 2ME (a reducing agent that disrupts disulfide bonds), with representative flow cytometry density plots and corresponding D) quantitative analysis (*n* = 5 per group). E) Differential interference contrast (DIC) microscopy images show morphological changes in SKOV3 cells after the above treatments; scale bar = 625 µm. F) PI+ cell percentage in IOSE‐80 cells after 24 h of glucose deprivation or 24 h of BAY‐876 (GLUT1 inhibitor) treatment, with representative flow cytometry density plots and corresponding G) quantitative analysis (*n* = 3 per group). H) DIC microscopy shows morphological changes in IOSE‐80 cells after the above treatments, along with PI single‐staining immunofluorescence images (red) and corresponding I) quantitative analysis (*n* = 3 per group); scale bar = 625 µm. J) PI+ cell percentage in CaoV3 cells after 24 h of glucose deprivation or 24 h of BAY‐876 treatment, with corresponding quantitative analysis (*n* = 3 per group). K) DIC microscopy shows no morphological changes in CaoV3 cells after 36 h of glucose deprivation, along with PI single‐staining immunofluorescence images (red) and corresponding L) quantitative analysis (*n* = 5 per group); scale bar = 625 µm. The *p* values were calculated using one‐way ANOVA. ns indicates no significance; *p < 0.05; **p < 0.01; ***p < 0.001; ****p < 0.0001. Data represent the mean ± SD of at least three independent experiments.

Glucose deprivation has been demonstrated to trigger disulfidptosis in *SLC7A11* ^high^ cancer cells.^[^ ^2^ ^]^ We conducted glucose deprivation experiments across multiple cell lines. Our results showed that SKOV3 cells displayed a significant increase in the percentage of propidium iodide‐positive (PI+) cells after 7 h of glucose deprivation (rising from a baseline of 0.96% to 4.44%, *p* < 0.05, **Figures** **5**A,B). We found that diamide (which induces disulfide bond accumulation) significantly enhanced glucose deprivation‐induced cell death (*p* < 0.001), whereas β‐mercaptoethanol (2ME, a reducing agent that breaks disulfide bonds) significantly alleviated cell death (*p* < 0.0001) (Figures 5C,D). Phase contrast microscopic observations revealed morphological changes and a reduction in cell density after 7 h of glucose deprivation, with diamide exacerbating this effect, while 2ME alleviated cell death and morphological changes (Figure 5E). In contrast, in IOSE‐80 cells (normal human ovarian epithelial cells), extending glucose deprivation to 24 h did not result in a significant increase in cell death (Figures 5F,G), and there were no statistically significant differences in cell death following treatment with 2ME or diamide (Figures 5H,I). In CaoV3 cells, glucose deprivation for up to 36 h did not increase cell death (Figures 5J–L). These findings suggest that SKOV3 cells are particularly sensitive to glucose deprivation, which leads to cell death, whereas IOSE‐80 and CaoV3 cells are glucose deprivation‐resistant, with minimal cell death observed, at least after short‐term glucose deprivation.

**Figure 6:**
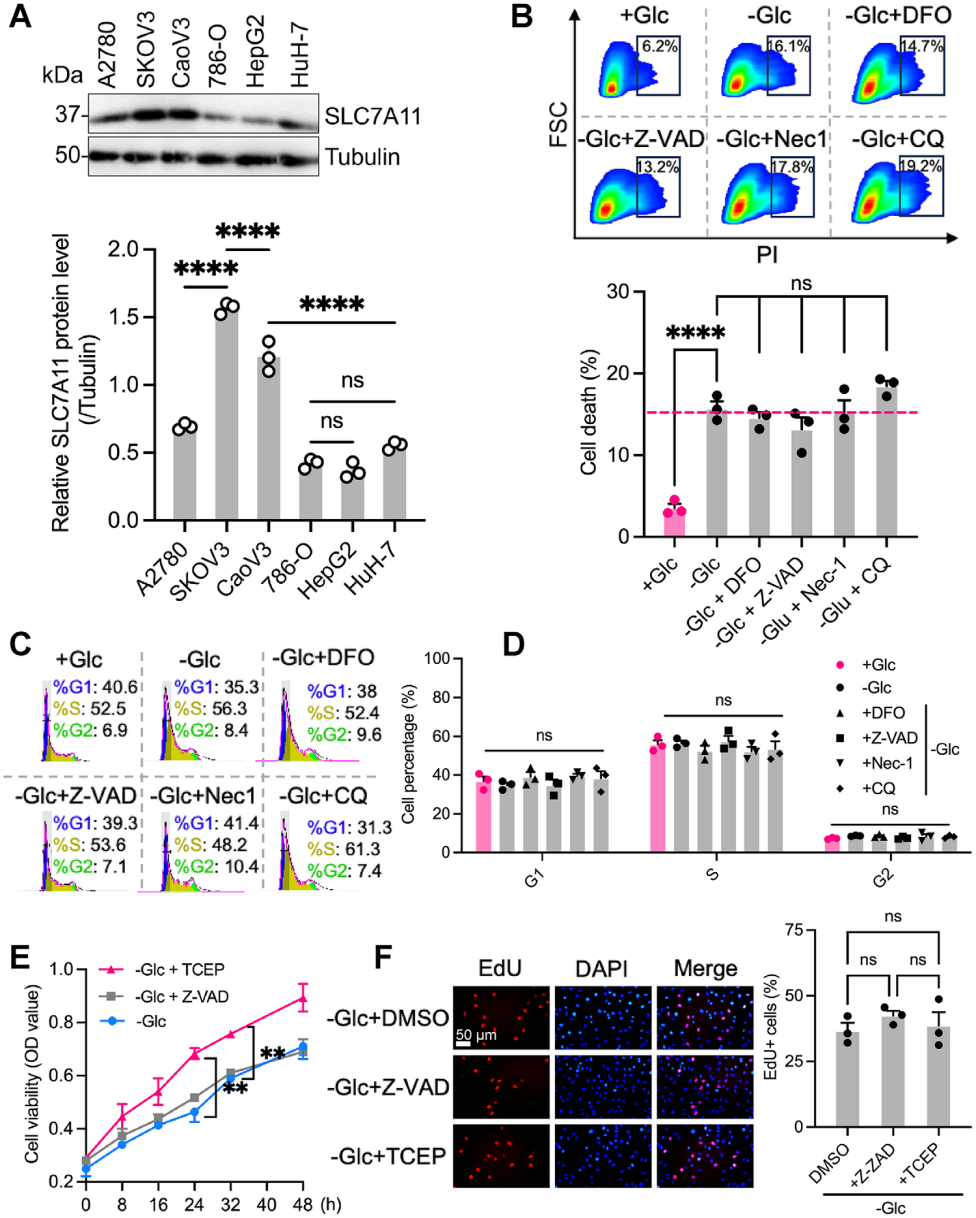
The occurrence of disulfidptosis was observed in glucose‐starved SLC7A11^high^ SKOV3 cells. A) SLC7A11 Western blots of different cell lines. B) Cell death in SKOV3 cells was measured by determining the proportion of propidium iodide (PI)‐positive cells after culturing them in glucose‐containing medium (+Glucose, Glc) or glucose‐free medium (‐Glc), with or without the indicated concentrations of DFO (100 µm), Z‐VAD‐FMK (Z‐VAD, 10 µm), necrostatin‐1s (Nec‐1s, 10 µm), and chloroquine (CQ, 25 µm) for ≈15 h. C) Cell cycle distribution of SKOV3 cells after indicated treatment for about 24 h with representative flow cytometry plots. D) Corresponding quantitative analysis of cell cycle distribution. E) Cell viability of SKOV3 cells was assessed using CCK‐8 assay after treatment with the indicated concentrations of Z‐VAD (10 µm) or TCEP (1 mm). F) Representative images of EdU staining and the percentage of SKOV3 cells positive for EdU were measured after indicated treatments for ≈24 h. The *p* values were calculated using a two‐tailed unpaired Student's t‐test in B and E, and one‐way ANOVA in D and F; ns means not significant; **p < 0.01; ****p < 0.0001. The data represent the mean ± SD of three independent repeats for all samples.

To further investigate whether glucose‐sensitive cell death in SKOV3 cells is associated with SLC7A11 expression levels, we examined the expression levels of *SLC7A11* in different tumor cell lines. The OV cell line SKOV3 exhibited increased *SLC7A11* expression (**Figure** **6**A). Upon glucose deprivation, *SLC7A11* ^high^ SKOV3 cells underwent cell death, which could not be reversed by inhibitors of various cell death pathways, including the ferroptosis inhibitor deferoxamine mesylate salt (DFO), the apoptosis inhibitor Z‐VAD‐FMK (Z‐VAD), the necroptosis inhibitor necrostatin‐1 (Nec‐1), or the autophagy inhibitor chloroquine (CQ) (Figure 6B). Although a slight shift in the cell cycle distribution was observed, the differences were not statistically significant (Figures 6C,D). The addition of the reducing agent TCEP relieved cell death induced by glucose starvation in *SLC7A11* ^high^ SKOV3 cells without influencing their proliferative abilities (Figures 6E,F). Hence, this cell death type in *SLC7A11* ^high^ SKOV3 cells is resistant to traditional cell death inhibitors, but can be ameliorated by reducing agents that inhibit disulfide reactions. Notably, these alterations did not substantially affect cell cycle progression or proliferative capacity.

**Figure 7:**
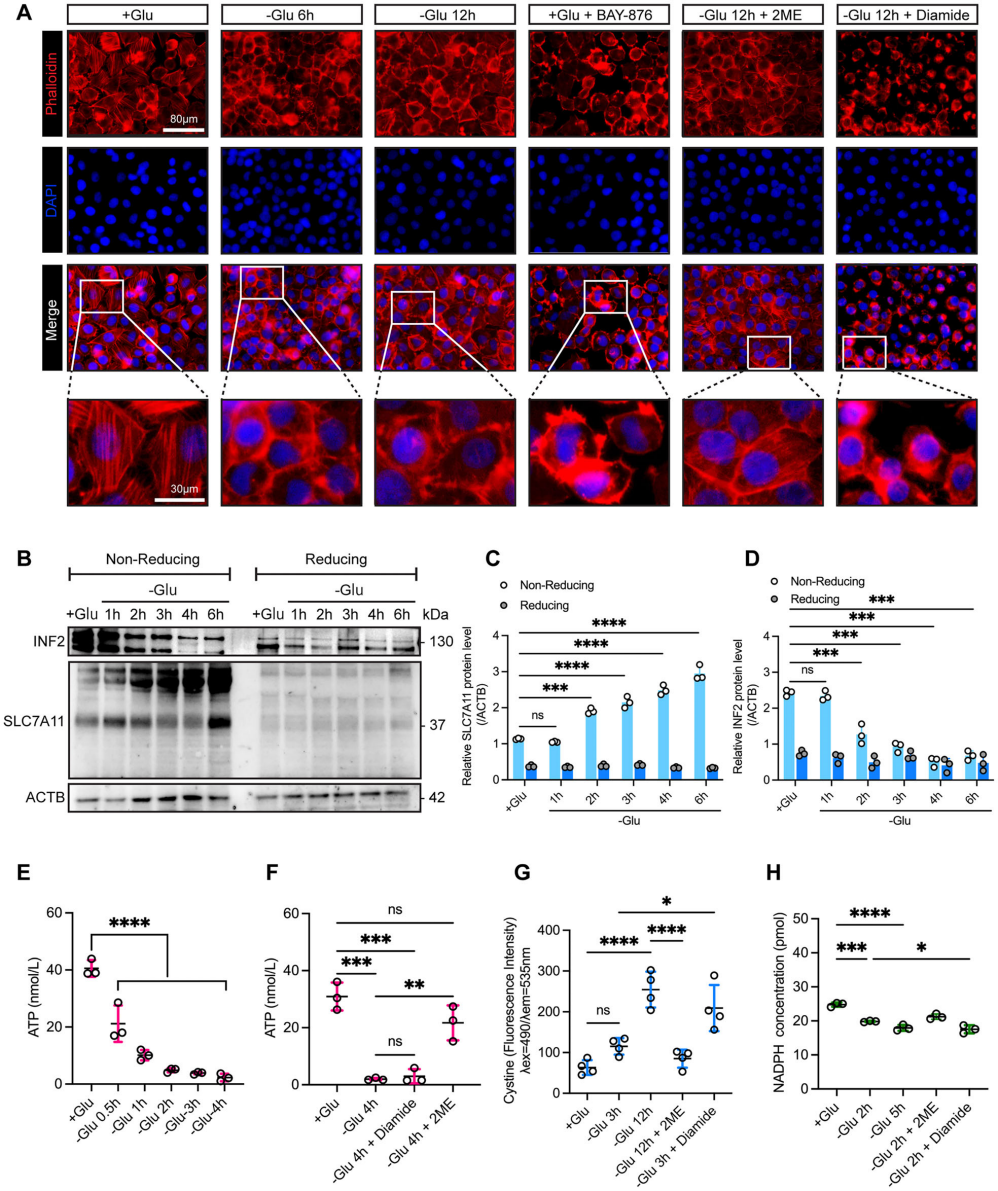
Disulfidptosis in SKOV3 Cells. A) Phalloidin staining (red) was used to observe changes in the cell cytoskeleton morphology of SKOV3 cells following glucose deprivation and corresponding treatments. B) Non‐reducing and reducing Western blot analyses were performed to examine the effect of glucose deprivation on the migration and expression of SLC7A11 and INF2 proteins in SKOV3 cells, with representative Western blot images. Corresponding quantitative analysis is shown in (C) and (D), based on grayscale densitometry. E,F) The impact of glucose deprivation and corresponding treatments on ATP production in SKOV3 cells was assessed. G) Cystine uptake in SKOV3 cells was measured under glucose deprivation conditions. H) NADPH levels in SKOV3 cells were determined following glucose deprivation. The *p* values were calculated using a one‐way ANOVA; ns indicates no significant difference; *p < 0.05; **p < 0.01; ***p < 0.001; ****p < 0.0001. Data are presented as mean ± SD from at least three independent experiments.

Under normal glucose conditions, the cytoskeletons of SKOV3 cells were clearly visible (**Figure** **7**A). However, after 6 h of glucose deprivation, the cytoskeleton collapsed, which worsened as the deprivation period was prolonged. Treatment with BAY‐876 under normal glucose conditions resulted in a cytoskeletal collapse, similar to that observed under glucose deprivation. Treatment with 2ME after glucose deprivation significantly improved the cytoskeletal structure. Under short‐term glucose deprivation, non‐reducing western blotting revealed a gradual upshift in SLC7A11 and a decrease in INF2 band intensity, whereas no such changes were observed under reducing conditions (Figures 7B–D). Short‐term glucose deprivation significantly reduced ATP levels (Figure 7E), increased cystine accumulation (Figure 7G), and led to marked NADPH depletion (Figure 7H) in OV cells. Treatment with the reducing agent 2‐ME reversed ATP loss and cystine accumulation (Figures 7F,G), highlighting the redox‐dependent nature of these changes. In contrast, diamide exacerbated cystine accumulation under glucose starvation without affecting ATP levels, and further depleted NADPH (Figures 7G,H), supporting its role in promoting disulfidptosis.

### 2.6 SLC7A11 Drives Disulfidptosis and Cytoskeletal Collapse via INF2‐Dependent Metabolic Stress

**Figure 8:**
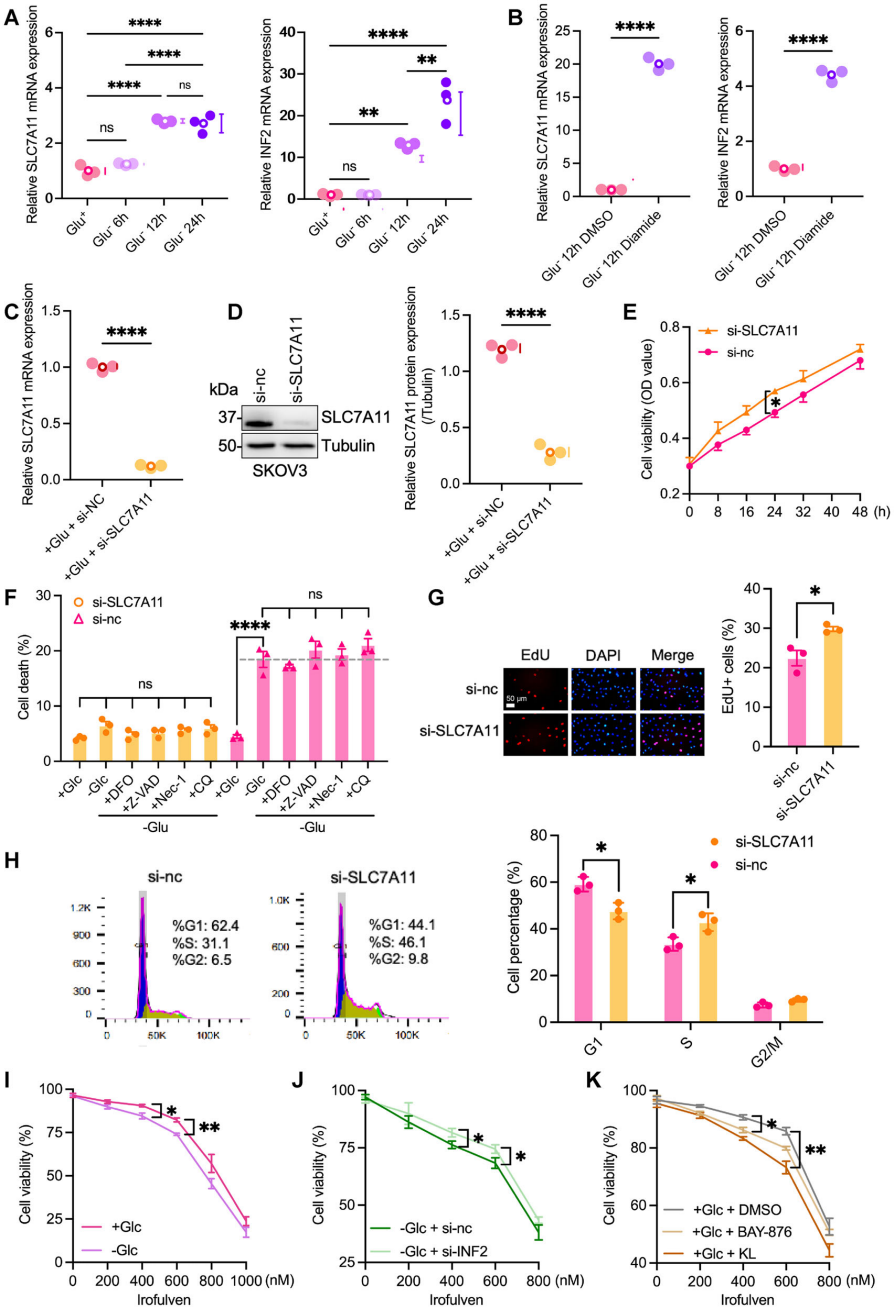
The effect of SLC7A11 and INF2 knockdown on SLC7A11^high^ SKOV3 cells. A) qRT‐PCR analysis showing the effect of different durations of glucose deprivation on the expression of SLC7A11 and INF2 mRNA. B) The effect of Diamide (a disulfide bond inducer) and the reducing agent 2ME on the mRNA expression of SLC7A11 and INF2 under glucose deprivation conditions. C) Knockdown efficiency of SLC7A11 by small interfering RNA (siRNA) assessed by qRT‐PCR. D) SLC7A11 Western blots in SKOV3 cells transfected using control vector (si‐nc) or si‐SLC7A11. E) The cell viability of SKOV3 cells was evaluated using the CCK‐8 assay after culturing in glucose‐free (‐Glc) medium and transfecting with either the si‐nc or si‐SLC7A11. F) Cell death in SKOV3 cells was quantified by determining the proportion of PI‐positive cells after culturing in glucose‐free (‐Glc) medium for 8 h and transfecting with either the si‐nc or si‐SLC7A11 for 24 h. G) The representative images of EdU staining and the percentage of EdU‐positive SKOV3 cells were evaluated after culturing in glucose‐free (‐Glc) medium and transfecting with either the si‐nc or si‐SLC7A11 for 24 h. H) Cell cycle distribution of SKOV3 cells culturing in glucose‐free (‐Glc) medium and transfection with either the si‐nc or si‐SLC7A11 for 24 h. I) The cell viability of SKOV3 cells was assessed using the CCK‐8 assay after culturing in the specified medium and transfection with either the si‐nc or si‐SLC7A11, in the presence or absence of indicated concentrations of Irofulven for a duration of 24 h. J) The cell viability of SKOV3 cells was evaluated after culturing them in the designated medium with either the si‐nc or si‐INF2, in the presence or absence of indicated concentrations of Irofulven for 24 h. K) The cell viability of SKOV3 cells was evaluated following culture in the specified medium and treatment with DMSO, KL‐11743 (5 µm), or BAY‐876 (5 µm), in the presence or absence of varying concentrations of Irofulven for a duration of 24 h. The *p* values were calculated using a two‐tailed unpaired Student's t‐test in B, C, D, E, G, H, I, and J, and the one‐way ANOVA in A, F, and K; ns means not significant; *p < 0.05; ** *p* < 0.01; ***p < 0.001; ****p < 0.0001. The data represent the mean ± SD of three independent repeats for all samples. The Western blots were repeated independently at least twice, and yielded similar results.

Building on the observation that glucose deprivation alters the redox‐dependent migration of SLC7A11 and INF2, we next investigated their transcriptional regulation. Quantitative reverse transcription polymerase chain reaction (qRT‐PCR) analysis showed no significant change in mRNA levels after short‐term glucose deprivation (6 h), but a marked upregulation of both *SLC7A11* and *INF2* after prolonged deprivation (12–24 h) (**Figure** **8**A), suggesting a potential feedback loop driving disulfidptosis in *SLC7A11* ^high^ cells. Consistently, diamide‐induced oxidative stress increased their mRNA expression (Figure 8B). Knockdown of *SLC7A11* in SKOV3 cells confirmed that disulfidptosis primarily occurs in *SLC7A11* ^high^ cells (Figures 8C–F). Notably, *SLC7A11* knockdown reduced disulfidptosis under glucose starvation and enhanced cellular proliferation, as demonstrated by increased EdU‐positive cells (Figure 8G) and a higher proportion of S‐phase cells accompanied by reduced G1‐phase cells (Figure 8H). These results indicate that *SLC7A11* regulates both disulfidptosis and cell cycle progression. The sensitivity of SKOV3 cells to irofulven was enhanced during glucose deprivation, potentially by inducing disulfidptosis (Figure 8I). Silencing *INF2* expression via transfection with si‐INF2 reduced the vulnerability of *SLC7A11* ^high^ SKOV3 cells to irofulven, which aligns with the predictions of drug responsiveness (Figure 8J). Previous studies have indicated that GLUT inhibition can directly initiate disulfidptosis.^[^ ^45^, ^46^ ^]^ Indeed, inhibitors of GLUT, such as BAY and KL, heightened the sensitivity of cells to irofulven, linking GLUT inhibition to disulfidptosis‐mediated therapeutic vulnerability (Figure 8K).

**Figure 9:**
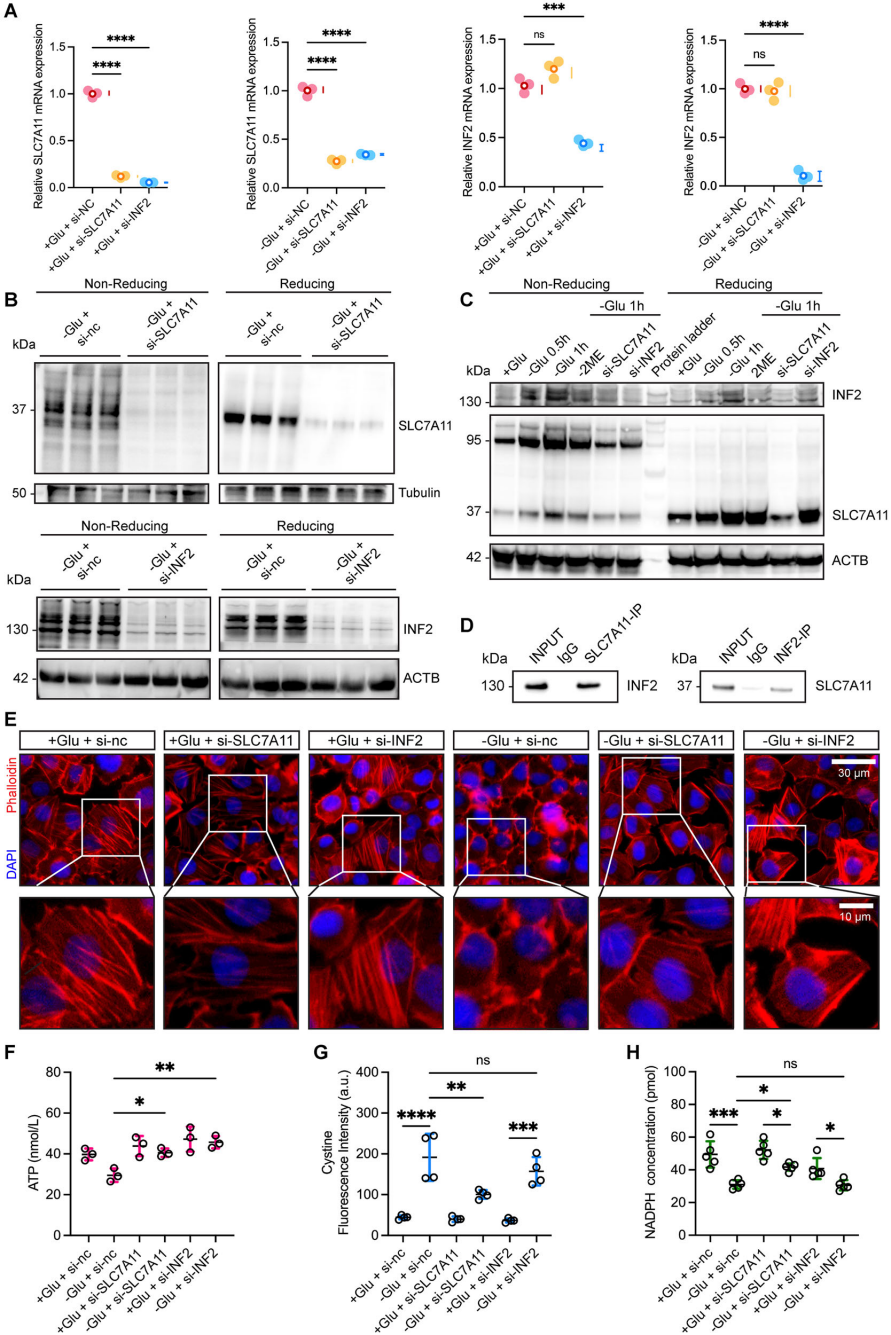
Knockdown of SLC7A11 and INF2 Abolishes Disulfidptosis Induced by Glucose Deprivation. A) qRT‐PCR analysis showing the effect of silencing SLC7A11 and INF2 on the expression of their respective mRNAs under normal glucose and glucose deprivation conditions. B) Non‐reducing and reducing Western blot analyses to assess the knockdown efficiency of SLC7A11 and INF2 under glucose deprivation. C) Western blot showing the impact of silencing SLC7A11 and INF2 on the protein expression of their respective targets. D) Co‐IP analysis demonstrating the protein‐protein interaction between SLC7A11 and INF2. E) Phalloidin staining (red) of the cell cytoskeleton in SKOV3 cells following glucose deprivation and knockdown of SLC7A11 and INF2, showing changes in cytoskeletal morphology. F) ATP production in SKOV3 cells following silencing of SLC7A11 or INF2. G) Cystine uptake capacity in SKOV3 cells following silencing of SLC7A11 or INF2. H) NADPH levels in SKOV3 cells after silencing of SLC7A11 or INF2. The *p* values were calculated using a one‐way ANOVA; ns indicates no significant difference; *p < 0.05; **p < 0.01; ***p < 0.001; ****p < 0.0001. The data represent the mean ± SD from at least three independent repeats for all samples. Western blots were repeated independently at least twice, yielding similar results.

To clarify the regulatory relationship between *SLC7A11* and *INF2*, we performed siRNA‐mediated knockdown under both normal and glucose‐deprived conditions. Silencing *INF2* effectively downregulated the mRNA expression of *SLC7A11*, whereas silencing *SLC7A11* did not significantly affect *INF2* mRNA expression (**Figure** **9**A). Knockdown efficiency at the protein levels was validated under both reducing and non‐reducing conditions during glucose deprivation (Figure 9B). At the protein levels, *SLC7A11* knockdown reduced INF2 protein expression under both reducing and non‐reducing conditions, while *INF2* silencing decreased SLC7A11 protein only under non‐reducing conditions (Figure 9C). This discrepancy between transcript and protein levels suggests that post‐translational mechanisms, possibly involving redox‐sensitive stabilization, contribute to SLC7A11‐INF2 regulation. Co‐immunoprecipitation (Co‐IP) assays confirmed the physical interaction between SLC7A11 and INF2 (Figure 9D), supporting their functional interplay in the execution of disulfidptosis. Functionally, knockdown of either *SLC7A11* or *INF2* had no observable effect on the cytoskeleton under normal glucose conditions. However, under glucose deprivation, silencing either *SLC7A11* or *INF2* both preserved cytoskeletal integrity, with the most prominent effect observed upon silencing *SLC7A11* (Figure 9E). These results showed that *SLC7A11* overexpression is required for disulfidptosis‐mediate cytoskeletal collapse, and that *INF2* likely acts downstream by modulating actin filament dynamics. To elucidate the metabolic mechanisms underlying these phenotypes, we assessed key indicators of cellular energy homeostasis. *SLC7A11* knockdown significantly increased intracellular ATP levels, reduced cystine uptake, and restored NADPH levels under glucose deprivation (Figures 9F–H). This suggests that *SLC7A11* contributes to metabolic stress by driving cystine overload and redox imbalance. In contrast, *INF2* silencing selectively elevated ATP without significantly altering cystine uptake or NADPH, indicating that *INF2* primarily contributes to disulfidptosis via ATP preservation and cytoskeletal (F‐actin) remodeling.

### 2.7 The *SLC7A11‐INF2* Axis Coordinates Disulfidptosis, Tumor Migration, and ROS Dynamics Under Glucose Deprivation

**Figure 10:**
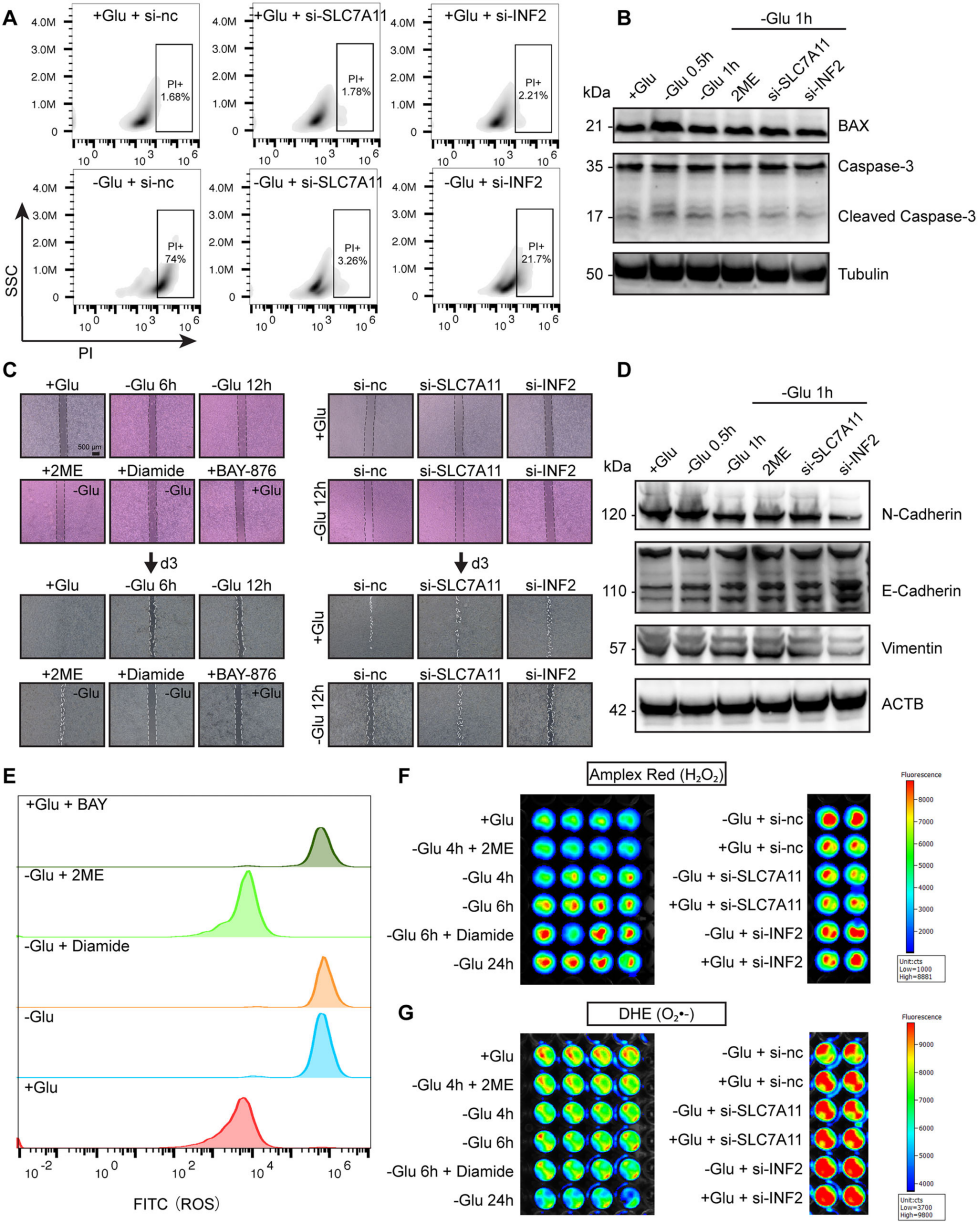
The SLC7A11‐INF2 Axis Affects Tumor Cell Migration and ROS Production. A) Flow cytometry analysis of PI staining in SKOV3 cells under glucose deprivation and following silencing of SLC7A11 and INF2, showing the percentage of PI+ cells, with representative flow density plots. B) Western blot analysis showing the effect of glucose deprivation and silencing of SLC7A11 and INF2 on apoptosis‐related proteins. C) Transwell migration assay assessing the impact of glucose deprivation and silencing of SLC7A11 and INF2 on the migratory capacity of SKOV3 cells (Day 1 and Day 3 after scratch). D) Western blot analysis showing the effect of glucose deprivation and silencing of SLC7A11 and INF2 on migration‐related proteins. E) Flow cytometry analysis of ROS levels in SKOV3 cells using ROS‐FITC fluorescence, showing the mean fluorescence intensity (MFI). F) Amplex Red assay for H2O2 detection in SKOV3 cells after transfection (*n* = 2 per group) and under different glucose deprivation treatments (*n* = 4 per group), showing the fluorescence intensity. G) Detection of superoxide anion levels in SKOV3 cells using DHE staining after transfection (*n* = 2 per group) and under different glucose deprivation treatments (*n* = 4 per group), with corresponding fluorescence intensity analysis. Western blots were repeated independently at least twice, yielding similar results.

We next investigated how the *SLC7A11‐INF2* axis modulates disulfidptosis, migration, and redox dynamics under glucose deprivation. Flow cytometric analysis revealed a significant increase in the percentage of PI+ SKOV3 cells subjected to glucose deprivation and silencing of *SLC7A11* or *INF2*, both partially rescued this phenotype, although *SLC7A11* knockdown had a stronger protective effect (**Figure** **10**A). Western blot analysis showed no changes in apoptosis markers, supporting that the cell death was non‐apoptotic and consistent with disulfidptosis (Figure 10B). Scratch wound assays demonstrated that under normal glucose, *INF2* knockdown moderately suppressed cell migration, while *SLC7A11* silencing had no evident effect (Figure 10C), suggesting *INF2* supports basal motility, likely via actin remodeling and mitochondrial fission. Under glucose deprivation, SKOV3 migration was markedly impaired. *SLC7A11* knockdown rescued this defect, while *INF2* knockdown failed to do so (Figure 10C), despite alleviating cytoskeletal collapse (Figure 9E). Consistently, *INF2* knockdown reduced mesenchymal markers N‐cadherin and vimentin, and increased E‐cadherin expression, indicating EMT suppression (Figure 10D).

We then assessed redox regulation by measuring reactive oxygen species (ROS). Total ROS levels were significantly increased under glucose deprivation, GLUT inhibition (BAY), and diamide treatment, while the reducing agent 2‐ME reversed this accumulation (Figure 10E). Amplex Red assays showed a time‐dependent rise in hydrogen peroxide (H_2_O_2_) levels under glucose deprivation, which was effectively attenuated by *SLC7A11* knockdown but not by *INF2* knockdown (Figure 10F). Conversely, DHE assays showed a decline in superoxide (O₂⁻•) under glucose deprivation or diamide treatment, while *INF2* knockdown maintained relatively high O₂⁻• levels (Figure 10G), possibly reflecting mitochondrial superoxide leakage due to impaired mitochondrial fission. Together, these findings indicate that *SLC7A11* knockdown suppresses disulfidptosis by restoring redox balance through reduced cystine uptake and NADPH depletion, thereby preserving cell viability and migration. In contrast, *INF2* knockdown alleviates disulfidptosis, likely through ATP preservation and cytoskeletal stabilization, but fails to reduce H₂O₂ accumulation or restore migration. These results highlight the distinct but complementary roles of the *SLC7A11–INF2* axis in coordinating disufidptosis, tumor migration, and redox signaling under metabolic stress.

### 2.8 The GLUT1 Inhibitor BAY‐876 Induces Disulfidptosis in *SLC7A11* ^high^ OV Cells in Primary and Liver Metastatic Tumors by Modulating the *SLC7A11*‐*INF2* Axis

**Figure 11:**
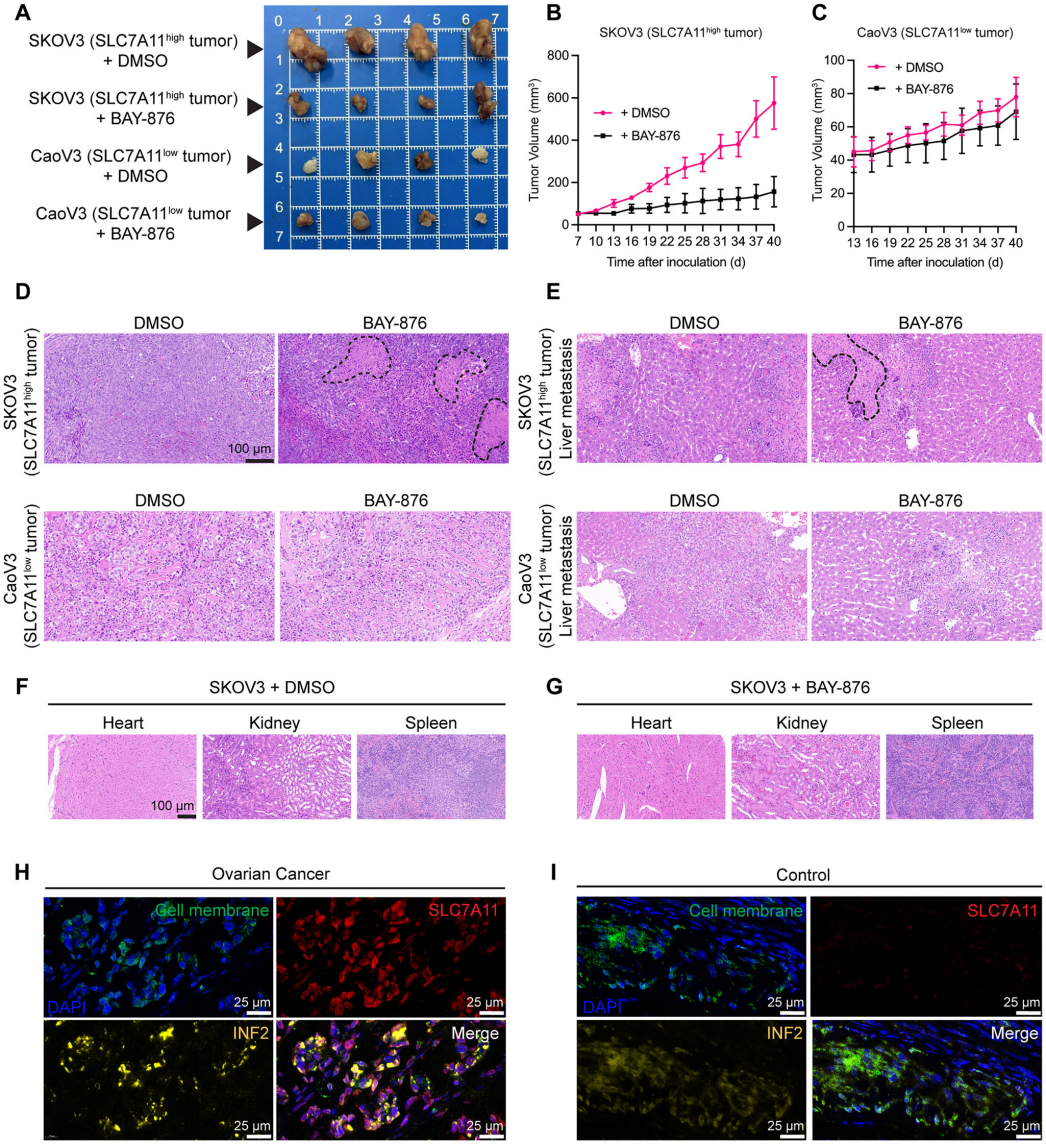
GLUT1 Inhibitor BAY‐876 Induces Disulfidptosis in SLC7A11^high^ Cells and Inhibits Migration to Treat Tumors. A) Tumor size and morphology on Day 40 after subcutaneous tumor formation in mice, with representative images of tumors in different treatment groups. B,C) Tumor volume measurements recorded over time after intraperitoneal injection of DMSO or BAY‐876 every 2 days, starting one week post subcutaneous tumor inoculation. D,E) Hematoxylin and eosin (HE) staining of tumor tissues from ovarian cancer cell xenografts, with necrotic areas marked by black dashed lines. F,G) HE staining of heart, kidney, and spleen tissues from control and treatment groups following tumor modeling. H,I) Immunofluorescence imaging of SLC7A11‐INF2‐cell membrane expression in tumor tissues from ovarian cancer patients and healthy control ovaries. The *p* values were calculated using a two‐tailed unpaired Student's t‐test in (B); the data represent the mean ± SD from three independent repeats for all samples.

To investigate the in vivo efficacy of GLUT1 inhibition, human OV xenograft models were established by subcutaneous injection of *SLC7A11* ^high^ SKOV3 and of *SLC7A11^l^* ^ow^ CaoV3 cells into female nude mice. In vivo tumor growth was monitored starting from day 7 (SKOV3) or day 13 (CaoV3) through day 40. As shown in **Figure** **11**A, CaoV3 tumors exhibited significantly lower tumorigenicity than SKOV3 tumors. Repeated intraperitoneal injections (i.p.) of BAY‐876 markedly suppressed SKOV3 tumor growth compared to vehicle control (DMSO) (Figures 11B,C). In contrast, CaoV3 xenografts exhibited minimal response to BAY‐876, indicating that the therapeutic effect depends on *SLC7A11* expression and disulfidptosis induction. Histological examination using hematoxylin and eosin (HE) staining revealed extensive necrotic areas in both primary tumors and liver metastases of BAY‐876‐treated SKOV3 xenografts (Figure 11D,E). Importantly, no significant toxicity was observed in vital organs, including the heart, kidneys, and spleen, indicating the selectivity of BAY‐876 for tumor tissues (Figures 11F,G). Immunofluorescence staining of tumor tissues from patients with OV showed widespread SLC7A11 overexpression and focal INF2 overexpression, with partial colocalization of the two proteins (Figure 11H). These patterns were absent in benign ovarian cyst tissues (Figure 11I). Together, GLUT1 inhibition by BAY‐876 selectively induces disulfidptosis in *SLC7A11* ^high^ tumor tissues, including both subcutaneous tumors and liver metastases, through modulation of the *SLC7A11–INF2* axis. This strategy holds promise as a redox‐based therapeutic approach for aggressive OV subtypes characterized by high *SLC7A11* expression.

## 3 Discussion

Disulfidptosis presents a distinct paradigm of cell death involving disulfides and aberrant glucose metabolism and is increasingly acknowledged in the field of oncology. The involvement of disulfidptosis in tumorigenesis is the focus of intense research, highlighting DRGs as potential prognostic markers in cancers such as THCA,^[^ ^47^ ^]^ KIRC,^[^ ^48^ ^]^ and esophageal squamous cell carcinoma.^[^ ^49^ ^]^ To systematically assess the clinical and biological implications of disulfidptosis in various malignancies, we developed a DRG score prognostic model and conducted a comprehensive pan‐cancer analysis using TCGA data. We found that the DRG score was significantly associated with poor prognosis, as high scores were correlated with aggressive tumor features, such as enhanced cell cycle progression, EMT, and angiogenesis. Our work builds upon a growing body of TCGA‐based biomarker studies, particularly those led by Liu et al, which pioneered batch‐oriented, scalable approaches for pan‐cancer biomarker discovery. Single‐gene studies have profiled TRPM7, RAD51, and CENPA as diagnostic and prognostic markers,^[^ ^50^, ^51^, ^52^ ^]^ while their gene set‐based frameworks have dissected the pan‐cancer signatures of disulfidptosis, cuproptosis, mitochondrial repair, and sodium channel regulation.^[^ ^53^, ^54^, ^55^ ^]^ These studies established methodological standards for high‐throughput analysis and biomarker prioritization across tumor types. In contrast to previous bioinformatics‐only pipelines, our model not only recapitulates the established features of disulfidptosis, but also expands upon them by integrating immune profiling, metabolic signatures, drug response data, and experimental validation. For instance, our GSEA revealed significant enrichment of KRAS signaling in tumors with high disulfidptosis scores, a novel finding not reported in previous pan‐cancer disulfidptosis studies. This underscores the expanded sensitivity of our model to capture clinically actionable pathways and highlights the potential links between disulfidptosis and oncogenic signaling.

Among the DRG components investigated in this study, *SLC7A11* has emerged as a central regulator. *SLC7A11* is essential for ferroptosis and is highly expressed in certain tumors, such as primary gliomas and OV.^[^ ^56^, ^57^ ^]^ *SLC7A11* supports cellular antioxidant defense by promoting glutathione synthesis; however, under glucose‐limited conditions, it paradoxically induces disulfidptosis by accumulating disulfide stress. Additionally, GLUT1 regulates glucose homeostasis and peptide hormone secretion, and is expressed in OV tissues, although its precise role remains to be clarified.^[^ ^58^ ^]^ Our observations revealed that disulfidptosis occured under glucose‐deprived conditions in SKOV3 cells expressing high levels of *SLC7A11*. This form of cell death induced by GLUT inhibitors notably enhances the sensitivity of OV cells to therapeutic agents, shedding light on the potential mechanisms of OV proliferation, metastasis, and targeted therapy. In our analysis using CCLE data, OV exhibited the highest expression of INF2, —a formin involved in actin remodeling, among gynecological malignancies. Furthermore, we observed a positive correlation between INF2 expression and immune scores, including immune and ESTIMATE scores, in various cancers, such as KICH, LICH, OV, PCPG, and PRAD. Across numerous cancer types, *INF2* mRNA was associated with various immune cell types, including M1 macrophages, activated CD4+ memory T cells, CD8+ T cells, and γδ T cells, suggesting a broader immunomodulatory function for INF2 beyond its canonical cytoskeletal role. *SLC7A11*, a key modulator of disulfidptosis, has emerged as a metabolic checkpoint in the TME with broad implications for immune cell function and immunotherapy responses.^[^ ^59^ ^]^ By mediating cystine uptake and promoting glutathione biosynthesis, *SLC7A11* contributes to redox homeostasis in tumor cells; however, it limits cystine availability to T cells, thereby inducing T cell ferroptosis, exhaustion, and the upregulation of inhibitory receptors, such as PD‐1 and TIM‐3.^[^ ^60^ ^]^ In contrast, the inhibition or genetic ablation of *SLC7A11* in tumor cells restores T cell activity and enhances antitumor immunity. Moreover, interferon (IFN)‐γ secreted by activated CD8+ T cells can synergize with ATM activation induced by radiotherapy to suppress *Slc7a11* expression, triggering ferroptosis in tumor cells and amplifying therapeutic efficacy.^[^ ^60^, ^61^ ^]^ *SLC7A11* also plays an emerging role in shaping myeloid immunity. For example, SLC7A11‐targeted ferroptosis in tumor‐associated macrophages reduces M2 polarization and infiltration, improving the efficacy of anti‐PD‐L1 therapy.^[^ ^62^ ^]^ Extracellular vesicles (EVs) released by PKM2‐activated T cells can promote iron accumulation and ferroptosis in macrophages, enhancing migration and contributing to inflammatory pathologies, such as cancer and aortic aneurysm.^[^ ^63^ ^]^ Importantly, next‐generation CAR‐T cell therapies are now being engineered to overcome the hostile metabolic conditions of solid tumors,^[^ ^64^ ^]^ such as elevated ROS and lactate levels, which are often driven by high SLC7A11 expression.^[^ ^65^ ^]^ Strategies to enhance mitochondrial fitness and oxidative stress resistance are being explored to improve CAR‐T cell persistence and function. In parallel, tumor‐derived EVs targeting SLC7A11 or related oxidative stress markers have been shown to reprogram macrophages toward immunosuppressive phenotypes.^[^ ^66^ ^]^ These findings support our observations that INF2 expression and the DRG score are associated with immune cell infiltration and immunoregulatory features across cancers, suggesting that disulfidptosis may influence both tumor immunity and therapeutic response and should be considered in future immunotherapy strategies.

In this study, we observed a compensatory relationship between SLC7A11 and INF2 in coordinating cell survival and motility. Mechanistically, under glucose deprivation, knockdown of *SLC7A11* alleviated cytoskeletal collapse and partially restored migration by reducing cystine accumulation, preserving ATP and NADPH levels, and suppressing disulfidptosis. In contrast, *INF2* knockdown protected cytoskeletal integrity primarily through ATP maintenance but failed to restore migratory capacity. INF2 is localized at mitochondria–ER contact sites and plays a critical role in regulating mitochondrial fission, bioenergetics, and actin filament dynamics.^[^ ^67^ ^]^ Previous studies have shown that INF2 is frequently upregulated in tumors, where it promotes mitochondrial division by recruiting DRP1.^[^ ^31^ ^]^ Furthermore, FBXO7 mutants associated with endometrial cancer impair the ubiquitination and degradation of INF2, resulting in mitochondrial hyper‐division, enhanced proliferation and migration, and resistance to apoptosis.^[^ ^24^ ^]^ We speculate that *INF2* knockdown—or its competitive displacement by SLC7A11—impairs its localization at ER–mitochondria junctions, thereby disrupting mitochondrial fission and actin remodeling, ultimately limiting cell motility. This may also explain the mild inhibitory effect of *INF2* knockdown on migration under normal glucose conditions. Under glucose starvation, SLC7A11 may competitively interfere with INF2 function or positioning, further suppressing mitochondrial fission and EMT progression despite the partial restoration of cytoskeletal structure.

This study has some limitations. First, although the TCGA encompasses 33 types of cancer and is currently the most comprehensive molecular map of human cancer, it does not capture all aspects of cancer biology. In our study, we conducted a conservative pan‐cancer analysis of key DRGs such as *SLC7A11*, which is commonly overexpressed in several solid tumors. Although the TCGA data offers significant insights, they are limited in terms of the diversity of cancer types and the inclusion of non‐coding genomic features. Furthermore, batch effects introduced by differences in sample collection, sequencing platforms, and pre‐analytical variables can introduce confounding signals that may obscure biological interpretations. Additionally, substantial cohort heterogeneity, including varying sample sizes, demographic imbalances, and differences in tumor purity across cancer types, can skew associations and reduce model generalizability, as emphasized in recent evaluations of bulk transcriptomic datasets.^[^ ^68^, ^69^ ^]^ Moreover, the TCGA dataset provides limited coverage of rare cancers and lacks the temporal sampling needed for dynamic biomarker tracking. These limitations highlight the need for prospective validation beyond the TCGA database. Finally, our in vivo experimental validation was restricted to OV, with a limited number of samples. Although INF2 has emerged as a compelling downstream mediator of disulfidptosis, its precise role and interaction with SLC7A11 require confirmation in additional tumor types.

To address these limitations and enable dynamic biomarker validation, we launched the Discover‐Trial, a prospective multicenter observational study involving 1200 patients with cancers. This trial was designed to evaluate disulfidptosis‐related biomarkers in a real‐world clinical setting. Its key objective was to assess SLC7A11 methylation levels in circulating tumor DNA (ctDNA) as a potential surrogate marker for disulfidptosis activation. Liquid biopsy‐based technologies such as ctDNA mutation analysis and methylation profiling have demonstrated utility in the early detection and treatment monitoring of neuroblastoma and pancreaticobiliary and breast cancers.^[^ ^70^, ^71^, ^72^ ^]^ Given that SLC7A11 is frequently overexpressed in tumors and exhibits epigenetic regulation, its methylation status in ctDNA may serve as a dynamic indicator of disulfidptosis activity. Integrating such assays into clinical workflows could enable the noninvasive tracking of metabolic stress responses and patient stratification for targeted therapy. Future studies should extend our findings by validating the DRG signature in patient‐derived xenograft models.^[^ ^2^ ^]^ These models preserve the heterogeneity and microenvironmental features of human tumors and offer a clinically relevant platform for testing how the SLC7A11‐INF2 axis affects tumor behavior and drug sensitivity under glucose‐deprived conditions.

## 4 Conclusion

In this study, we successfully established a DRG scoring model that supports the prediction of clinical outcomes and drug responses across various cancer types. Our results revealed that elevated DRG scores were positively associated with both poor prognosis and aggressive cancer cell behavior. In particular, cancers with high DRG scores, such as those in *SLC7A11* ^high^ OV cells, tend to undergo disulfidptosis upon glucose deprivation. We also found that the suppression of *SLC7A11* and *INF2* expression effectively hindered disulfidptosis and decreased the vulnerability of *SLC7A11* ^high^ cells to glucose deprivation, an effect that could be counteracted by GLUT inhibitors. Furthermore, our study elucidated the roles of SLC7A11 and INF2 in regulating tumor progression, disulfidptosis, and migration. The complex interplay between these proteins and their impact on ROS production under stress conditions opens up new avenues for targeted cancer therapies. Further investigations of the molecular mechanisms underlying their actions are essential to develop strategies to manipulate this axis for cancer treatment.

## 5 Experimental Section

### Sample Collection

Tissue samples comprising six benign ovarian cysts and six ovarian carcinoma tissues were collected from patients at Zhongshan Hospital of Fudan University, following the decision for surgical intervention. The acquisition of tissues through clinical surgery and the conduct of this study adhered to the Declaration of Helsinki. All patients who participated in the study had a confirmed diagnosis of ovarian serous cystadenocarcinoma via pathological assessment and no history of autoimmune diseases, neoadjuvant chemotherapy, or radiotherapy before the surgical procedure. The ethical guidelines under which this study was conducted were sanctioned by the Research Ethics Committee of the Zhongshan Hospital of Fudan University (approval number: B2022‐457).

### Data Acquisition and Analysis

TCGA datasets were retrieved from the UCSC Xena platform (https://xenabrowser.net/datapages/). This platform is recognized as the most comprehensive database for cancer gene information and encompasses data on gene expression, copy number variations, and single‐nucleotide polymorphisms. The TCGA dataset used in this study for independent observations and validations included 8739 pan‐cancer cases from 32 tumor types, excluding samples of acute myeloid leukemia. 70% of the samples were randomly assigned to the training set (6484/9264) and the remaining 30% to the test set (2780/9264). Table S2 (Supporting Information) shows the details of the samples in the training and test sets involving specific tumors in the TCGA. Mutation information for tumor cell lines and data related to drug resistance were sourced from the Broad Institute CCLE database (https://portals.broadinstitute.org/ccle/). The IMvigor210 cohort provided the transcriptome information (http://research‐pub.gene.com/IMvigor27CoreBiologies), and the GSE78220 dataset contained transcriptomic information from patients with metastatic melanoma undergoing anti‐PD‐1 treatment. DRGs were obtained from previously published studies, as described in Table S3 (Supporting Information).^[^ ^2^ ^]^

### Development of a Prognostic Index Linked to DRGs

To analyze the DRGs, dimensionality reduction and prevention of overfitting during model development were achieved using the LASSO algorithm.^[^ ^73^, ^74^ ^]^ The LASSO regression was maintained with ten‐fold cross‐validation and selected the optimal regularization parameter using the one‐standard‐error rule, which identifies the most parsimonious model within one standard deviation of the minimum cross‐validated mean squared error.^[^ ^75^ ^]^ Details of the model implementation, including random seed setting and internal cross‐validation parameters, are provided in the “Methods” Section (Supporting Information). Subsequently, the Cox proportional hazards regression model was used to evaluate the relationship between DRG expression and clinical endpoints, including DSS, OS, and PFI, within the training cohort from the TCGA pan‐cancer studies. Using this Cox regression model for DRGs, the coefficient β for each DRG was derived, enabling the computation of each patient's risk score using the formula:

(1) $$DRGs\;riskscore=\sum_{i=1}^{n}\beta i*Expi$$

where *i* denotes a specific gene within the prognostic signature, *Expi* is the expression level of gene *i*, and *βi* is the coefficient for gene *i* obtained from the multivariate Cox regression analysis. Following the zero‐mean normalization of the risk scores, patients were stratified into high‐ and low‐risk groups. The discriminative performance of the model was quantified using Harrell's concordance index (C‐index) to assess the accuracy of the survival risk stratification. Variables significantly correlated with survival were identified iteratively through cross‐validation. Final parameter estimation was performed using the Breslow method to handle the tied event times.

### Assessment of Biological Processes via z‐Score Methodology

The z‐score methodology, as described by Lee et al.,^[^ ^76^ ^]^ was employed to assess the expression of genes and their impact on specific biological pathways, such as angiogenesis, EMT (GO):0001837), the cell cycle, and initial markers of DRGs.^[^ ^77^, ^78^ ^]^ To compute the z‐scores for angiogenesis, EMT, cell cycle, and DRGs for each gene set, the GSVA package was utilized in R. This method facilitates the identification of pathway activities through unique gene expression characteristics.^[^ ^79^ ^]^

### Creating a Pan‐Cancer Nomogram

The correlations among DRGs, clinical features, and survival outcomes were explored using both univariate and multivariate Cox regression analyses.^[^ ^80^ ^]^ Utilizing the R package rms, a nomogram with a calibration curve was constructed. In addition, a multivariate regression framework was established to examine links between the DRG risk scores and the clinical attributes of each cancer type. Scores were assigned to the different levels of each influencing factor, and these scores were summed to obtain the total predictive score. The probability of prediction was then derived from the aggregated score. The prognostic efficacy of the nomogram and the DRG risk‐scoring model was assessed using time‐dependent ROC curve analysis, from which the area under the curve was computed.

### Analysis of Pathways in Gene Modules and GSEA

To explore the functional relationships of genes within the identified modules in depth, the ClusterProfiler package was utilized in R for gene annotation purposes.^[^ ^81^ ^]^ Both the GO and KEGG databases were harnessed to identify related functional categories. Pathways determined to be significant were those with *p*‐values and q‐values below the threshold of 0.05 in both the GO and KEGG enrichment analyses. Additionally, we conducted a GSEA to examine the connection between cancer traits and the processes of disulfidptosis, thereby identifying pertinent cellular signaling pathways in the context of cancer.

### Evaluation of the TME and Immune Cell Infiltration Analysis

The infiltration of immune cells and the characteristics of the TME were assessed using the ESTIMATE algorithm in R, based on differences in gene expression.^[^ ^82^ ^]^ This method allowed the calculation of immune, stromal, and estimated scores across various samples. Furthermore, the CIBERSORT algorithm was applied to RNA sequencing (RNA‐seq) data from patients with 33 cancer types across different subgroup classifications to infer the proportion of various infiltrating cells and analyze their associations with gene expression and immune cell types.^[^ ^83^, ^84^ ^]^ Additionally, the TISIDB online platform (http://cis.hku.hk/TISIDB) was utilized to investigate the associations between gene expression profiles and immunomodulators such as chemokines, immunosuppressive agents, immune stimulants, and MHC molecules.^[^ ^85^ ^]^

### Assessment of Drug Sensitivity

Constructed using 60 distinct cancer cell types from the Cancer Research Center of the National Cancer Institute, the CellMiner database is a crucial tool for evaluating the efficacy of anticancer agents. For this study, sensitivity data for drugs tested on NCI‐60 cells and RNA‐seq gene expression data were acquired. Correlation analyses were conducted to explore relationships between gene expression and sensitivity to commonly used chemotherapeutic agents.

### Evaluation of the TMB, MSI, and NEO

The TMB was calculated as the overall count of non‐synonymous mutations, including base changes, additions, or deletions observed per million bases. Quantification of the TMB involved dividing the number of mutations that alter the amino acid sequence by the entire length of the protein‐coding regions, following which the mutation rate and variation per exon for each tumor sample were determined. The MSI scores for patients in the TCGA database were sourced from a previously published study.^[^ ^86^ ^]^ Meanwhile, the DRGsMHCpan v3.0 tool was used to analyze the NEO values for each patient.^[^ ^87^ ^]^

### Forecasting Immunotherapy Outcomes

Participants were stratified into responders and non‐responders based on clinical outcomes. The differences in response rates and OS between patients with high and low DRGs expression were compared.

### Evaluation of Mutation Status in Tumors

Assessment of the gene copy number involved identifying both heterozygous and homozygous amplifications or deletions. SCNAs exhibiting a high frequency were characterized by alterations in more than 5% of the cases examined. The association between the SCNA and gene expression levels was evaluated using Pearson's correlation coefficient. Analysis of the exclusivity among tumor suppressor genes, oncogenes, or driver genes across various cancers was performed using the R package DISCOVER.^[^ ^88^ ^]^ Data on somatic mutations from the TCGA were sourced from the Genomic Data Commons Portal (https://portal.gdc.cancer.gov/). Based on the approach of Zhao et al., the truncating mutations were defined as Frame_Shift_Del, Frame_Shift_Ins, and nonsense mutations.^[^ ^89^ ^]^ Using Maftools, significantly mutated genes were identified within DRG‐defined risk groups, focusing on genes that were mutated more than 30 times in any group. The proportions of mutations were statistically examined using a one‐sided z‐test and a two‐sided chi‐square test, with the significance level set at *p* < 0.05.

### Cell Lines and Cell Culture

Human ovarian adenocarcinoma cell lines SKOV3, CaoV3, and A2780, and human hepatocellular carcinoma cell lines HepG2, Hep3B, and HuH‐7 were acquired from the American Type Culture Collection (ATCC, Manassas, VA, USA). Normal human ovarian epithelial cells (IOSE‐80) were kindly provided by Li Hong's laboratory of Shanghai Jiao Tong University. The supplier conducted mycoplasma screening tests to verify that the cell lines were free from contamination. In addition, the results of the vendor‐performed short tandem repeat profiling were cross‐checked with the International Cell Line Authentication Committee database to confirm the authenticity and correct identification of each cell line. For cell culture, the cells were grown in Dulbecco's modified Eagle medium (DMEM; Thermo Fisher Scientific) supplemented with 10% fetal bovine serum (FBS; Biological Industries, 04‐001‐1ACS) and a mixture of penicillin (100 IU mL^−1^) and streptomycin (100×) (Pricella; PB180120) in an incubator at 37 °C with a humidified atmosphere containing 5% CO_2_. During the glucose deprivation studies, the cells were cultured in glucose‐depleted DMEM (11966025; Thermo Fisher Scientific) supplemented with 10% FBS.

### Reagents

The following compounds were used to inhibit various cell death pathways and glucose metabolism: deferoxamine mesylate salt (DFO; D9533), a ferroptosis inhibitor; chloroquine (CQ, C6628), an autophagy suppressor; and BAY‐876 (SML1774), a GLUT1 antagonist (all supplied by Sigma–Aldrich). The following reagents were obtained from MedChemExpress: necrostatin‐1 (Nec‐1, HY‐15760), which impedes necroptosis; KL‐11743 (HY‐145597), which blocks GLUT 1–4; Z‐VAD‐FMK (HY‐16658B), an apoptosis inhibitor; and irofulven (HY‐14429), a DNA alkylating agent. Diamide (10465‐78‐8) was also purchased from Sigma–Aldrich. Last, Tris‐(2‐carboxyethyl)‐phosphine (TCEP, T2556) and β‐mercaptoethanol (2ME, 31350010), two reductants utilized to alleviate disulfide stress, were purchased from Thermo Fisher Scientific.

### Small Interfering RNAs (siRNAs) and Transfection

To inhibit *SLC7A11* and *INF2* expression, antisense oligonucleotides modified with locked nucleic acids were used. GenePharma supplied siRNAs specific to *SLC7A11* (si‐SLC7A11), si‐INF2, and control sequences that do not target any specific genes. The following siRNA sequences were employed in this study: si‐SLC7A11, sense: 5′‐GGGUGGAACUCCUCAUAAUTT‐3′, antisense: 5′‐AUUAUGAGGAGUUCCACCCTT‐3′; si‐INF2, sense: 5′‐GGAGAUCACUUUCCUCGAUTT‐3′, antisense: 5′‐AUCGAGGAAAGUGAUCUCCTT‐3′; and negative control (si‐nc), sense: 5′‐UUCUCCGAACGUGUCACGUTT‐3′, antisense: 5′‐ACGUGACACGUUCGGAGAATT‐3′. To facilitate the efficient delivery of these siRNAs into cells, we used the Lipofectamine 2000 transfection reagent (CAS No. 11668500; Invitrogen).

### RNA Extraction and qRT‐PCR

RNA was extracted from SKOV3 cells using TRIzol reagent, and the RNA concentration was measured. Reverse transcription was performed using a SweScript All‐in‐One RT SuperMix for qPCR (One‐Step gDNA Remover, G3337‐50; Servicebio). qRT‐PCR was conducted using a Universal Blue SYBR Green qPCR Master Mix (G3326‐01, Servicebio). The primers used for qRT‐PCR are listed in **Table** **S4** (Supporting Information). Finally, gene expression normalization was performed using the 2^−ΔΔCT^ method.

### Non‐Reducing and Reducing Western Blotting, and Co‐IP Assays

Before treating the cell lysates, the protease inhibitor PMSF was added to the RIPA lysis buffer at a 1:100 ratio. A BCA kit (Solarbio; PC0020) was used to measure protein content in the supernatant. Subsequently, the lysate proteins were denatured by heating with 5× loading buffer (reducing loading buffer, G2013‐1ML; non‐reducing loading buffer, G2030‐1ML; both from Servicebio) for 10 min. These denatured proteins were then separated using SDS‐PAGE (8–15% polyacrylamide gels) and subsequently transferred onto polyvinylidene difluoride membranes for western blotting. To block nonspecific binding, the membranes were incubated with 5% bovine serum albumin in TBST, followed by an overnight incubation with primary antibodies targeting tubulin (ab7291, 1:5000, Abcam), INF2 (ab245555, 1:2000, Abcam), ACTB (ab8227, 1:1000, Abcam), BAX (50599‐2‐Ig, 1:2000, Proteintech), caspase‐3 (19677‐1‐AP, 1:2000, Proteintech), cleaved caspase‐3 (82707‐13‐RR, 1:5000, Preteintech), N‐cadherin (22018‐1‐AP, 1:2000, Proteintech), E‐cadherin (20874‐1‐AP, 1:20000, Poteintech), Vimentin (60335‐1‐Ig, 1:4000, Proteintech), and SLC7A11 (ab207601, 1:1000, Abcam). After incubation with the primary antibodies, the membranes were washed with TBST and incubated with the corresponding secondary antibodies at a dilution of 1:5000. Fiji software was used for semi‐quantitative analysis. For the Co‐IP experiments, a COIP kit (Bes3011, Bersin Bio) was used to verify the protein interactions between INF2 and SLC7A11.

### Cell Death, Cell Viability, and Cell Cycle Assays

SKOV3, CaoV3, and IOSE‐80 cells were seeded into 12‐well plates and cultured for 24 h before treatments to evaluate cell death. After treatment with media with or without the specific drugs, the cells were collected into 1.5 mL microtubes. Cells were then washed with PBS and resuspended in cold PBS containing 1 µg mL^−1^ PI. PI‐positive (dead) cells were assessed via flow cytometry using a BD Accuri C6 cytometer (BD Biosciences). To assess cell viability, ≈5000–10000 cells were plated in each well of 96‐well plates one day before initiating treatment. The spent medium was then replaced with fresh culture medium containing 10% Cell Counting Kit‐8 (CCK‐8) reagent (CK04, Dojindo). The plates were then incubated for 1 h in a cell incubator, and the absorbance was measured at 450 nm using a microplate reader (Molecular Devices, USA). Cell viability was quantified as follows: cell viability (%) = [(absorbance of the tested compound − absorbance of the blank)/(absorbance of the control − absorbance of the blank)] × 100. For cell cycle analysis, SKOV3 cells were cultured with or without the specific treatment, then collected, and fixed in cold 70% ethanol overnight. Afterward, the cells were stained with 0.5 mL of PI/RNase Staining Buffer (550825, BD Biosciences) for 15 min at room temperature. FlowJo v10 software was used for the analysis.

### Scratch Wound Healing Assay

SKOV3 cells were seeded in 12‐well plates and treated accordingly. Once the cells reached confluence, vertical lines were created on the surface of the wells using a pipette tip perpendicular to the bottom of the culture plate. On the day of line marking, the medium was replaced with a serum‐free culture medium. Images were captured using a brightfield microscope on the day of marking, as well as at 3 days after line marking.

### 5‐Ethynyl‐2′‐Deoxyuridine (EdU) Incorporation Assay

A CellLight EdU Apollo567 In Vitro kit (C10310‐1; RiboBio) was used to evaluate cellular proliferation. Briefly, SKOV3 cells were seeded into 96‐well plates and treated with or without specific pharmacological agents. Next, 50 µm of EdU labelling medium was added to each well. The plates were then incubated at 37 °C for 2 h. Afterward, the cells were fixed using 4% paraformaldehyde, treated with glycine, permeabilized, and exposed to Apollo solution (RiboBio). Hoechst 33342 was used for nuclear staining, followed by a series of washes. Images were then captured and analyzed using a fluorescence microscope (Axio Scope.A1, Zeiss, Oberkochen, Germany). The proportion of EdU‐positive cells relative to the total number of Hoechst‐positive cells was determined from three random fields in three replicate wells.

### ATP, Cystine, and NADPH Assays

ATP levels were measured using an enhanced ATP assay kit from Beyotime (S0027), and detection was performed using a luminometer. Cystine uptake was assessed using a Cystine Uptake Assay Kit (UP05, Dojindo). Special cystine‐free serum was used for cell culture. Fluorescence intensity was measured using a microplate reader at excitation and emission wavelengths of 485 and 535 nm, respectively. NADPH levels were measured using an enhanced NADP+/NADPH assay kit from Beyotime (WST‐8 method), and absorbance at 450 nm was detected.

### ROS, H_2_O_2_, and O₂⁻• Detection

ROS levels were measured using an ROS Assay Kit (Beyotime, S0033S), and fluorescence intensity was detected via flow cytometry. H_2_O_2_ levels in the cell supernatants were determined using an Amplex Red Assay (Beyotime, ST010‐5 mg) at an excitation wavelength of 571 nm and emission wavelength of 585 nm. Superoxide anions (O₂⁻•) were assessed using dihydroethidium (DHE), a fluorescent probe, to detect live cells. DHE emits blue fluorescence at excitation and emission wavelengths of 370 and 420 nm, respectively. Upon oxidation, DHE binds to RNA or DNA, producing red fluorescence at an excitation wavelength of 300 nm and an emission wavelength of 610 nm. Fluorescence images were captured using a fluorescence imaging system, and the fluorescence intensity was quantified using a microplate reader for statistical analysis.

### Immunofluorescence Analysis

Patient‐derived, formalin‐fixed, paraffin‐embedded tissue sections (5 mm thick) were prepared for immunofluorescence analysis. Co‐staining of SLC7A11, INF2, and the cell membrane was performed using a fluorescence kit (RecordbioBiological Technology, Shanghai, China) according to the manufacturer's guidelines. Briefly, the slides were deparaffinized in xylene and rehydrated in an ethanol series. Antigen retrieval was facilitated by microwaving the slides in citrate buffer (pH 6.0). The primary antibodies used were anti‐SLC7A11 (rabbit, 1:200; TD12509; Abmart, Shanghai, China) and anti‐INF2 (rabbit, 1:50; 20466‐1‐AP; Proteintech, Wuhan, China). Next, the cell membrane was stained using a green fluorescent probe (40725ES10, Yeasen, China) for 1 h at room temperature in a moisture‐controlled chamber. Changes in the cytoskeleton were detected using a CoraLite594‐labeled Cyclic Peptide Staining Kit (PF00003; Proteintech). Finally, cell nuclei were stained using 4′,6′‐diamidino‐2‐phenylindole (DAPI) before examination under a laser confocal microscope (Fluoview FV1000, Olympus, Tokyo, Japan).

### Subcutaneous Tumor Implantation, Intraperitoneal Drug Administration, and Sample Processing in OV Mouse Models

A total of 24 female nude mice (3–4 weeks old) were purchased from Youshu Biosciences (Shanghai, China) Co., Ltd., and divided into four groups, with six mice in each group. Subcutaneous tumor cells injection was performed using SKOV3 and CaoV3 cells (1 × 10^7^ cells/mouse). Tumor growth was monitored starting on day 7 post‐injection, and the tumor volume was recorded. Once tumors were established, BAY‐876 (3 mg kg^−1^) was dissolved in 100 µL of 40% DMSO‐physiological saline solution and administered via intraperitoneal injection. Mice were observed every 2 days for general health status and tumor size measurements. Mice were euthanized once tumor growth did not exceed 15 mm (in compliance with ethical guidelines). Tumors were harvested and imaged under a microscope. Additionally, heart, kidney, and spleen tissues were collected to assess potential drug toxicity and processed for HE staining. All animal experiments were reviewed and approved by the Institutional Animal Welfare and Ethics Committee of Youshu Biosciences (Shanghai) Co., Ltd. (approval number: YS‐m202412001) and conducted in compliance with the National and Institutional Guidelines for the Care and Use of Laboratory Animals. The study design, execution, and reporting followed the ARRIVE 2.0 guidelines.

### Statistical Analysis

Data were analyzed using R software (version 4.2.1) for database mining and bioinformatic exploration. Univariate survival analyses were used to calculate hazard ratios and 95% confidence intervals. Kaplan–Meier curves were used to depict the association between patient survival and gene expression levels. Gene expression levels were evaluated using the Wilcoxon test. GraphPad Prism software (version 10) was used to create visual representations of the experimental results, which were conducted in triplicate and presented as the mean ± standard deviation. Comparisons of normally distributed data were performed using Student's *t*‐tests, whereas the Mann–Whitney U test was applied for non‐normally distributed data. For multiple data comparisons, analysis of variance (ANOVA) with Dunnett's test was used, and *p* < 0.05 was considered statistically significant.

### Ethics Approval Statement

The ethical guidelines under which this study was conducted were sanctioned by the Research Ethics Committee of Zhongshan Hospital (Approval No.: B2022‐457). All animal experiments were reviewed and approved by the Institutional Animal Welfare and Ethics Committee of Youshu Biosciences (Shanghai) Co., Ltd. (approval number: YS‐m202412001).

## Conflict of Interest

The authors declare no conflict of interest.

## Author Contributions

Z.S., Q.Y., and L.H. contributed equally to this work and are co‐first authors. Z.S. and X.X. conceptualized the framework of the study. L.H. performed the bioinformatics analysis, and Q.Y. carried out the validation and formal analyses. J.H. provided guidance on the bioinformatics analysis, and D.L. supervised the experimental procedures and methodology. D.C. collected and managed clinical samples. B.Z. oversaw the project administration. Z.S. and J.X. conducted both the in vitro and in vivo experiments. L.W. contributed to experimental validation. Z.S. was responsible for the original draft preparation. X.X. (lead corresponding author) led the overall supervision of the study, coordinated cross‐disciplinary collaboration, and was responsible for manuscript reviewing, editing, data visualization, and proofreading.

## Supporting information



Supporting Information

Supplemental Table 1‐4

## Data Availability

The custom codes used for R analysis were obtained from the Bioconductor website (https://www.bioconductor.org/). All datasets involved in this study can be viewed in the Gene Expression Omnibus (GEO), the Cancer Genome Atlas (TCGA), and the Molecular Signature Database (MSigDB) (https://www.gsea‐msigdb.org/gsea/msigdb/), or the Data Availability sections of the corresponding publications. All other data supporting the findings of this study are available within the article and its Supplementary Information files or from the corresponding authors upon reasonable request.
