# Supplementary material for: Glucose Deprivation‐Induced Disulfidptosis via the SLC7A11‐INF2 Axis: Pan‐Cancer Prognostic Exploration and Therapeutic Validation
Source: Adv Sci (Weinh). 2025 Jul 25;12(39):e08556. doi: 10.1002/advs.202408556 (PMC12533382; doi:10.1002/advs.202408556)
Supplement: Supplementary file 1 — Supporting Information [file ADVS-12-e08556-s001.docx]

Supporting Information

**Glucose Deprivation-Induced Disulfidptosis via *SLC7A11-INF2* Axis: Pan-Cancer Prognostic Exploration and Therapeutic Validation**

Zhenyu Song^1†^, Lina Huang^4,5†^, Qiuming Yao^6,7†^, Dan Cui^1^, Jun Xie^3^, Leilei Wu^8^, Jianfeng Huang^9*^, Bo Zhai^1*^, Dan Liu^2*^, Xiao Xu^3*^

Figure S1. The disease specific survival (DSS), overall survival (OS), progression-free interval (PFI) of the 33 cancers in TCGA training set.


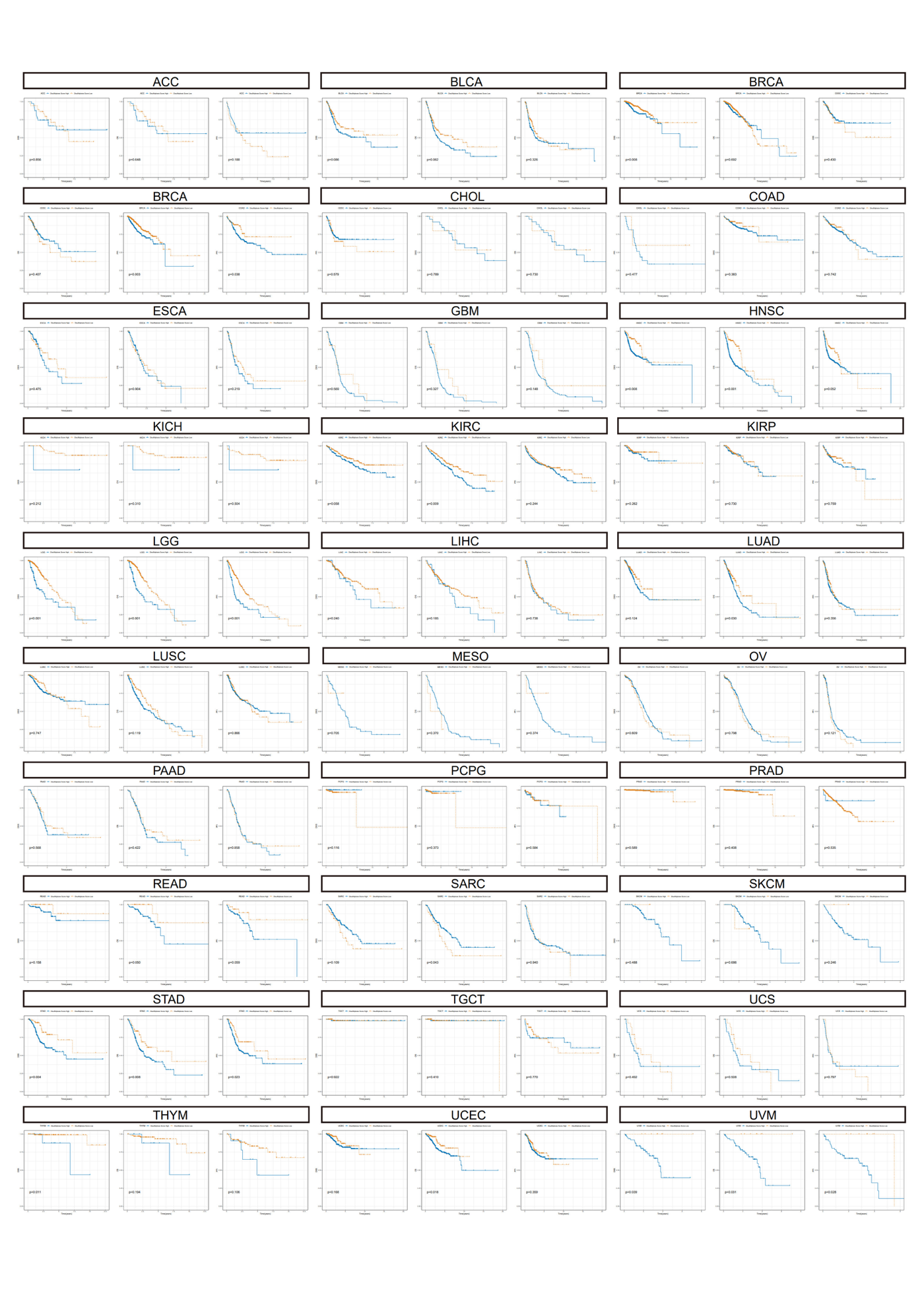


Figure S2. The correlation analyses were performed between the DRG score and angiogenesis z-score (A), as well as cell cycle z-score (B) in individual cancer types.


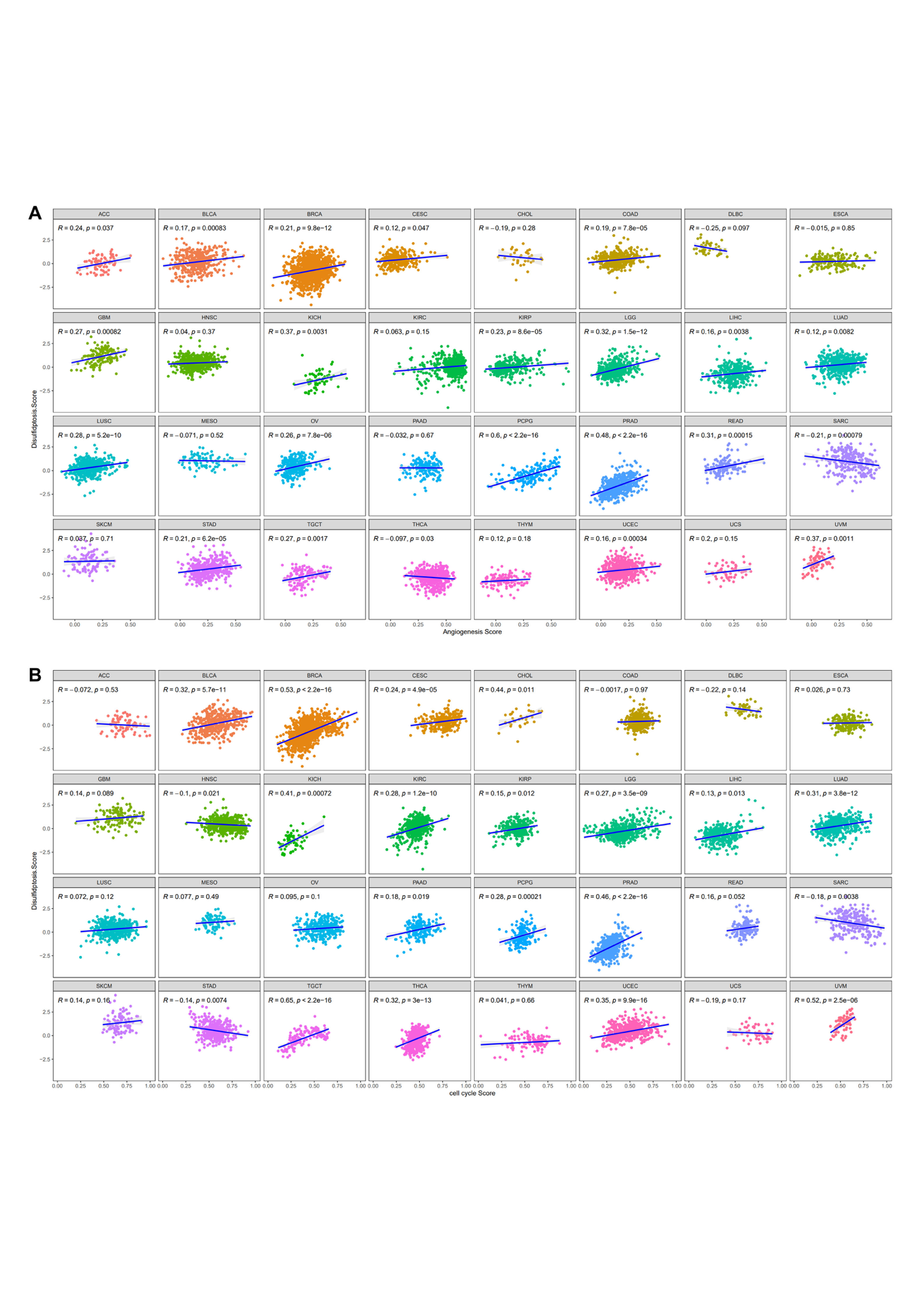


Figure S3. Expression levels analyses of hub DRGs. (A) The expression levels of hub DRGs were analyzed in TCGA cohorts, comparing tumor and normal tissue across 33 different types of tumors. (B) The expression levels of hub genes were assessed across various tumor cell lines using the Cancer Cell Line Encyclopedia (CCLE) database from the Broad Institute. * p < 0.05; ** means p < 0.01; *** means p < 0.001; **** means p < 0.0001; ns means not significant.


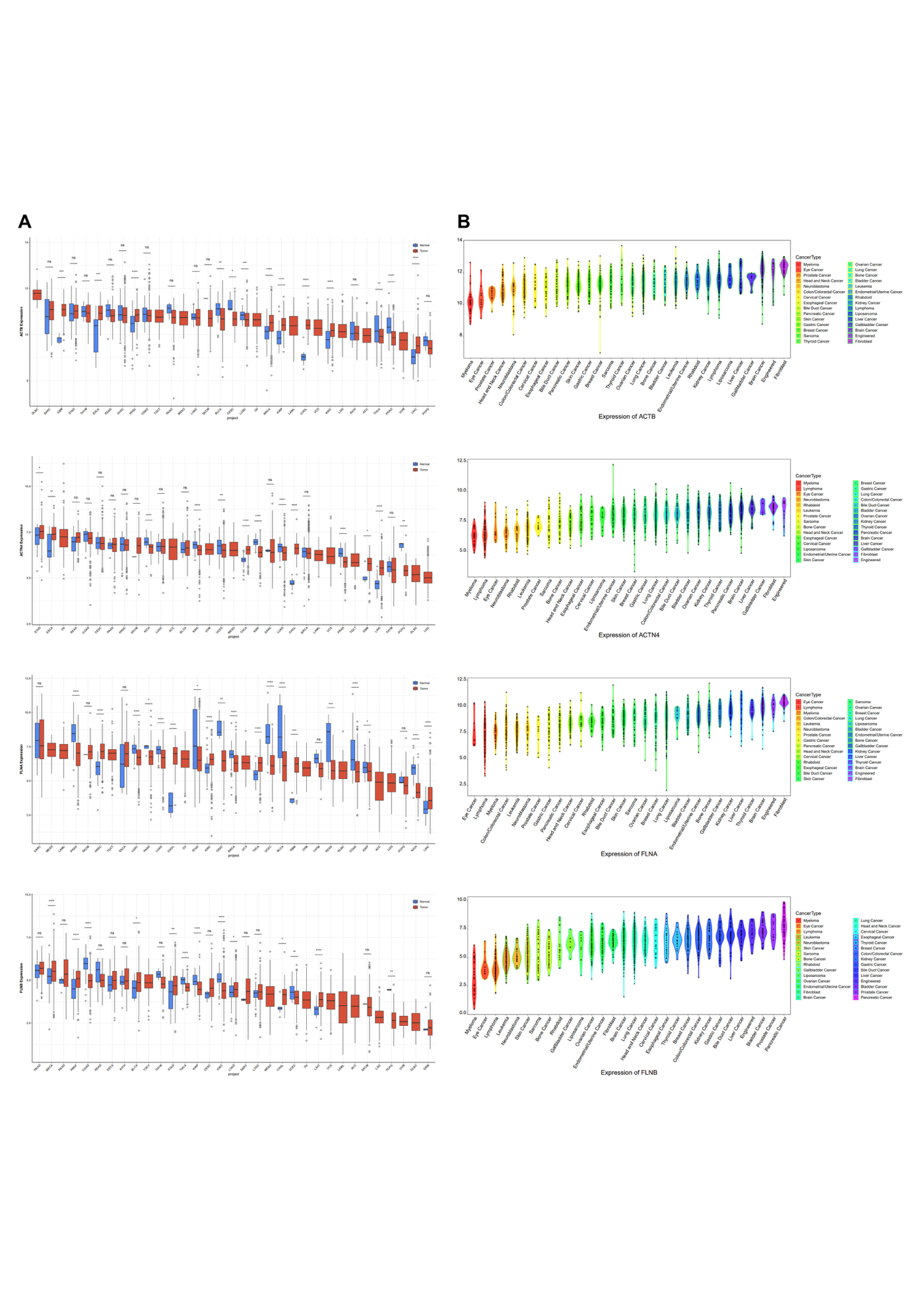


Figure S4. Correlation between the expression of hub DRGs and immune score, as well as tumor immune infiltration.


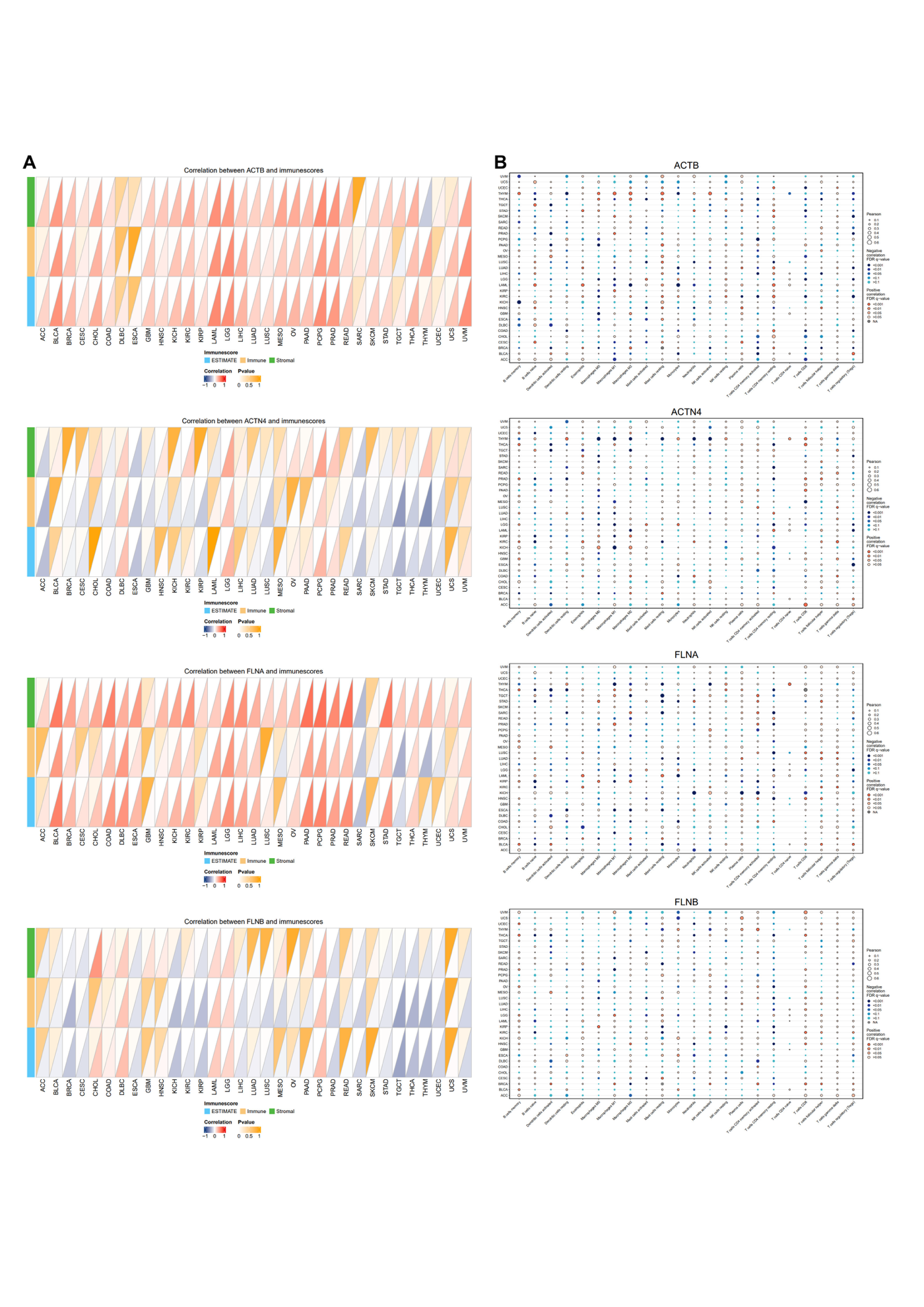


Figure S5. Correlation between the expression of INF2 and the tumor microenvironment across 33 types of tumors.


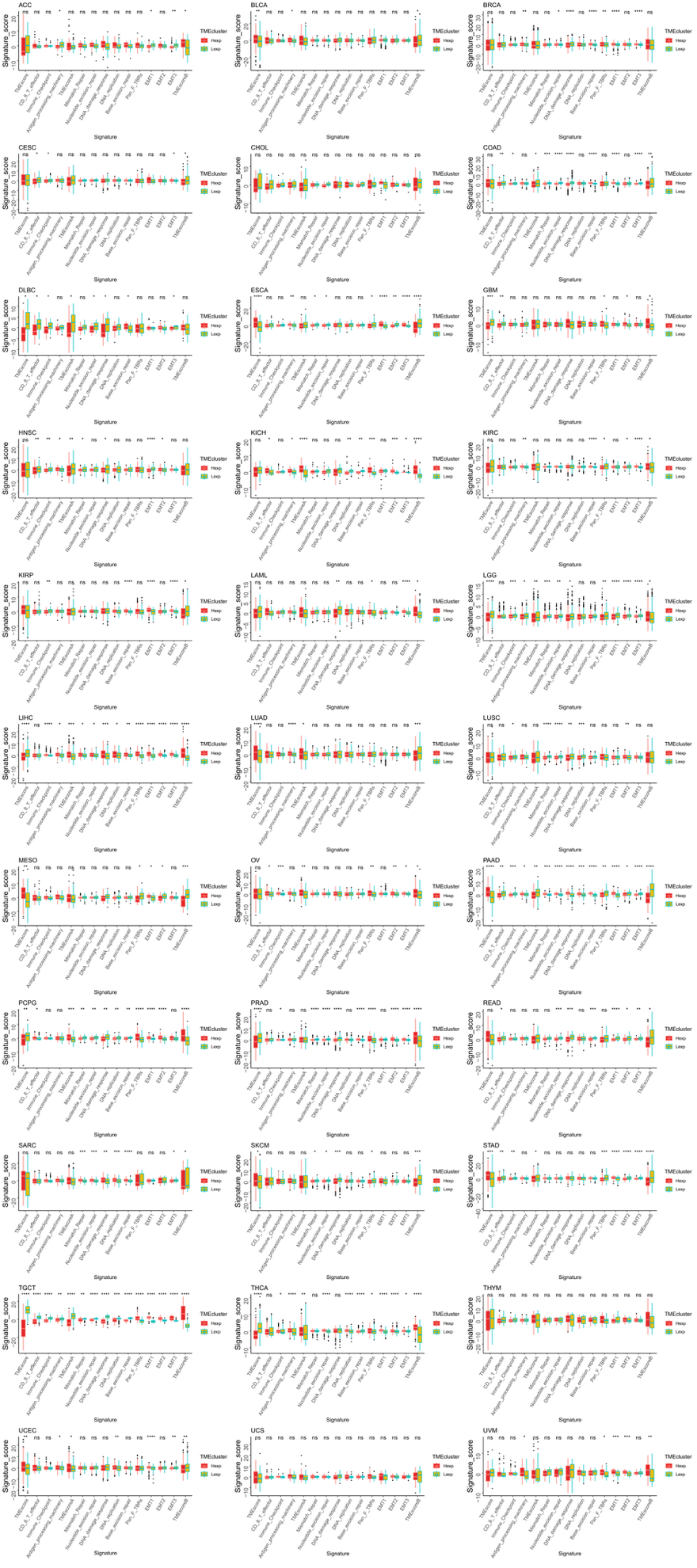


Figure S6. Efficacy of DRGs in predicting drug sensitivity.


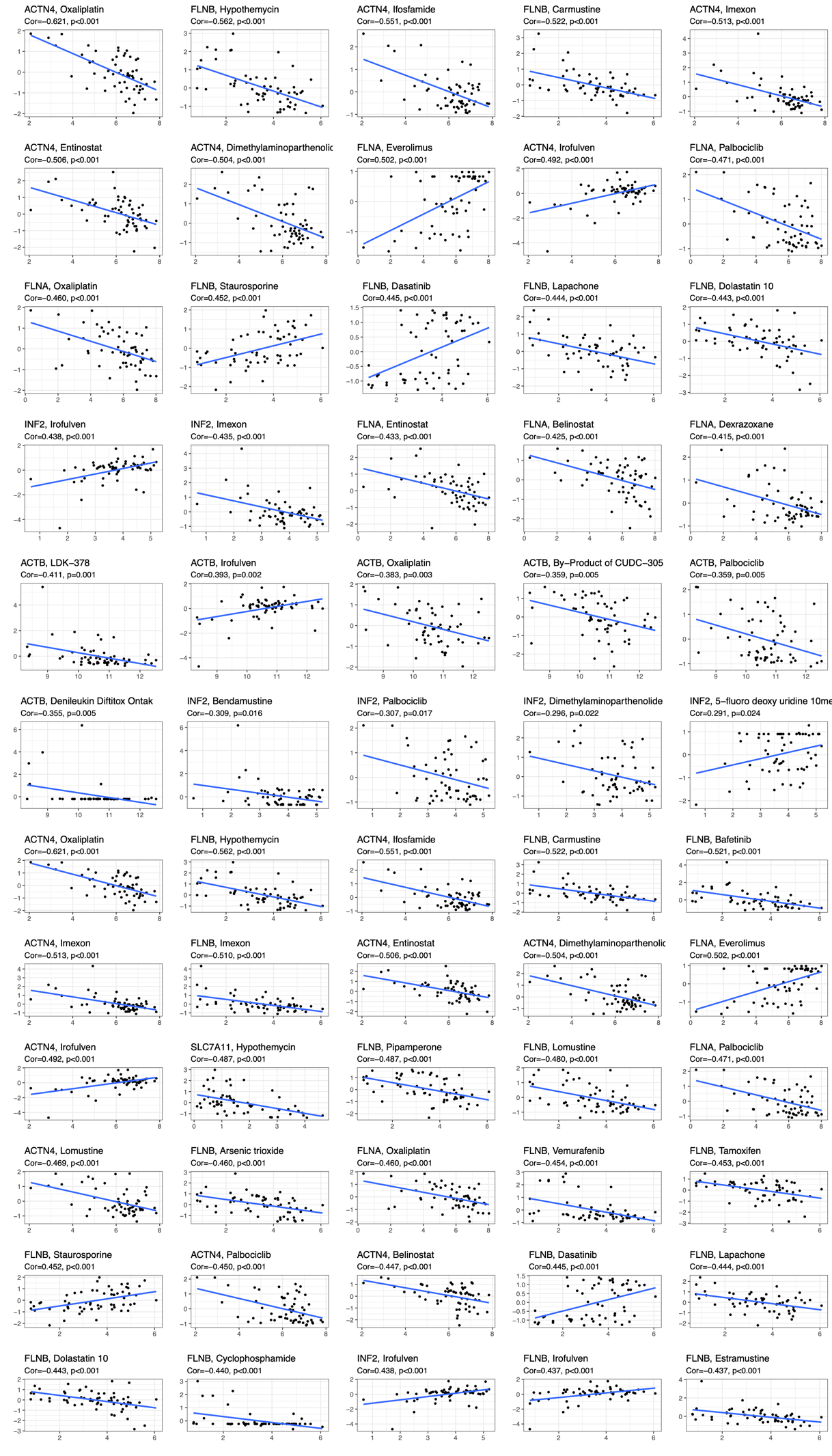


Figure S7. Correlation analysis of INF2 expression levels with key pathways in tumor regulation.


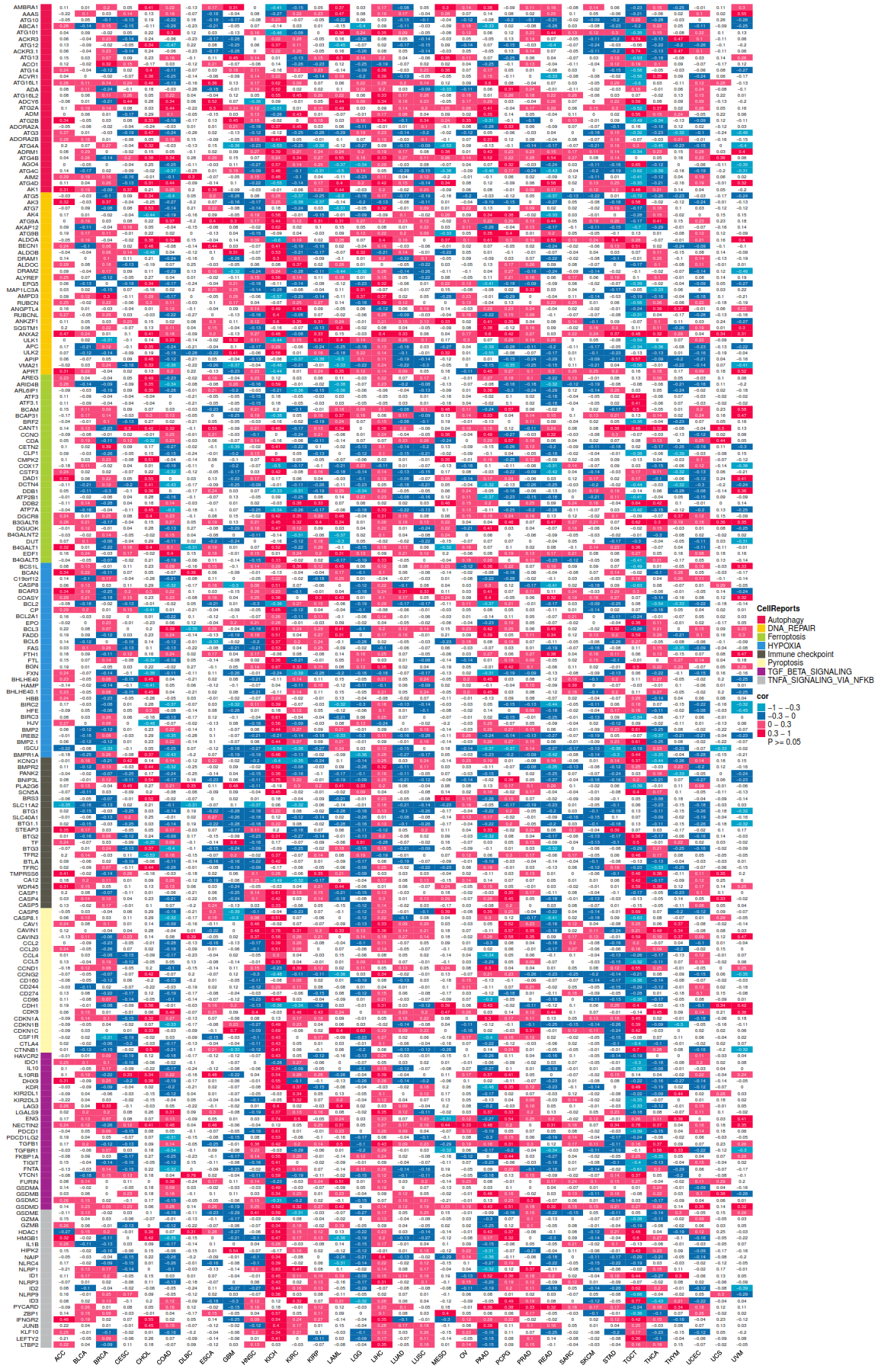


Figure S8. Correlation analyses between the expression of ACTB and crucial tumor regulatory pathways.


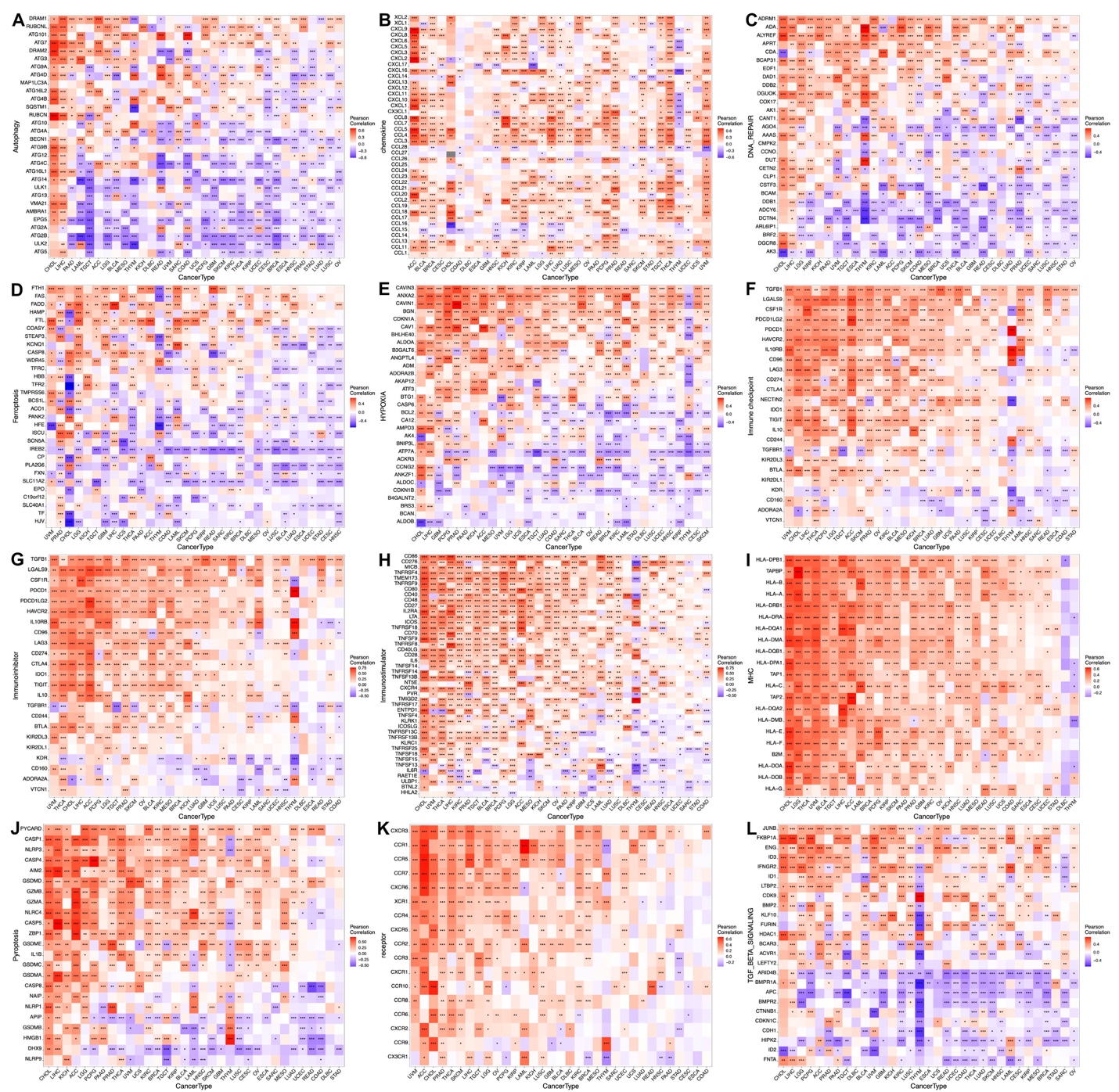


Figure S9. Correlation analyses between the expression of ACTN4 and crucial tumor regulatory pathways.


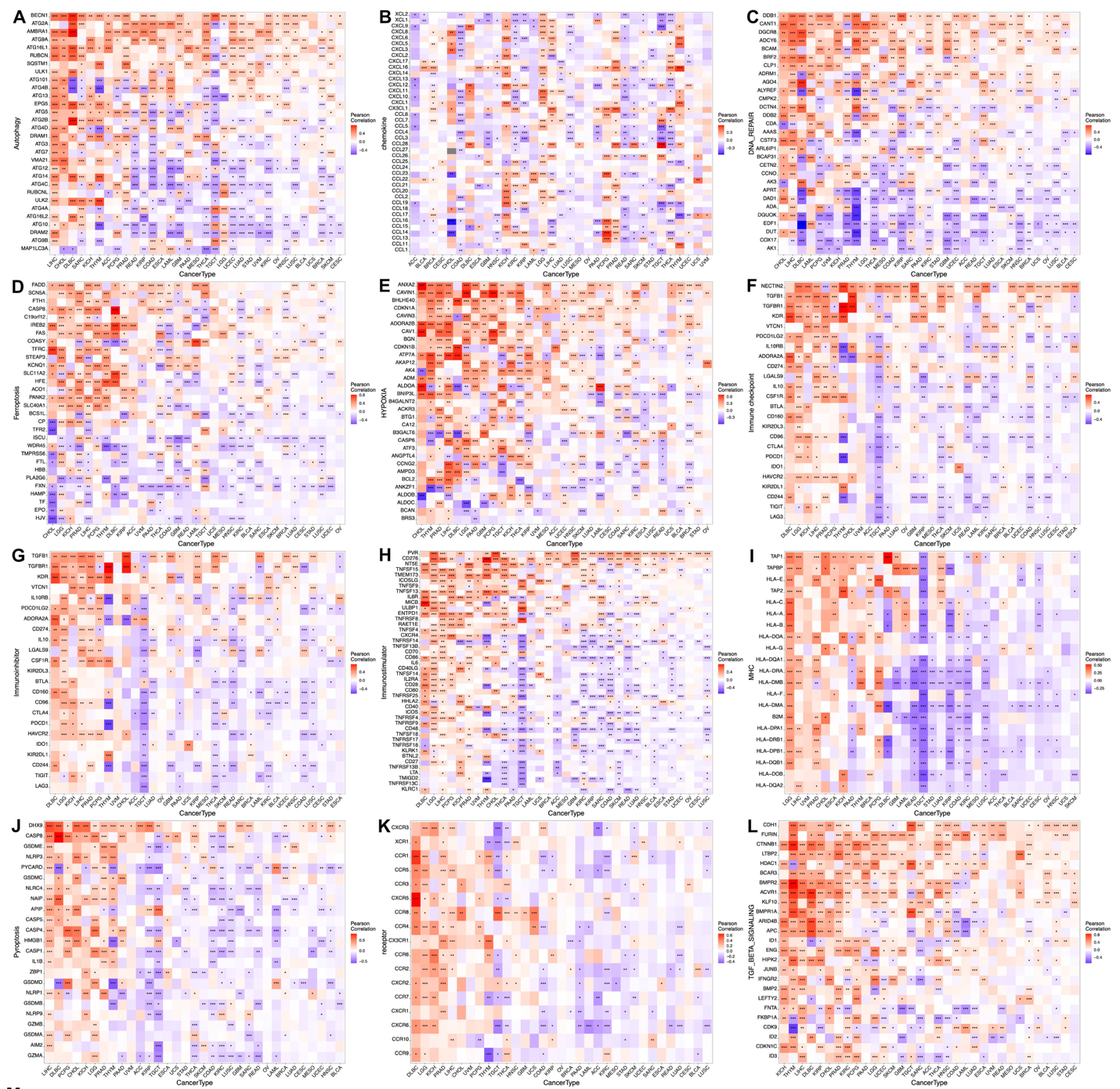


Figure S10. Correlation analyses between the expression of FLNA and crucial tumor regulatory pathways.


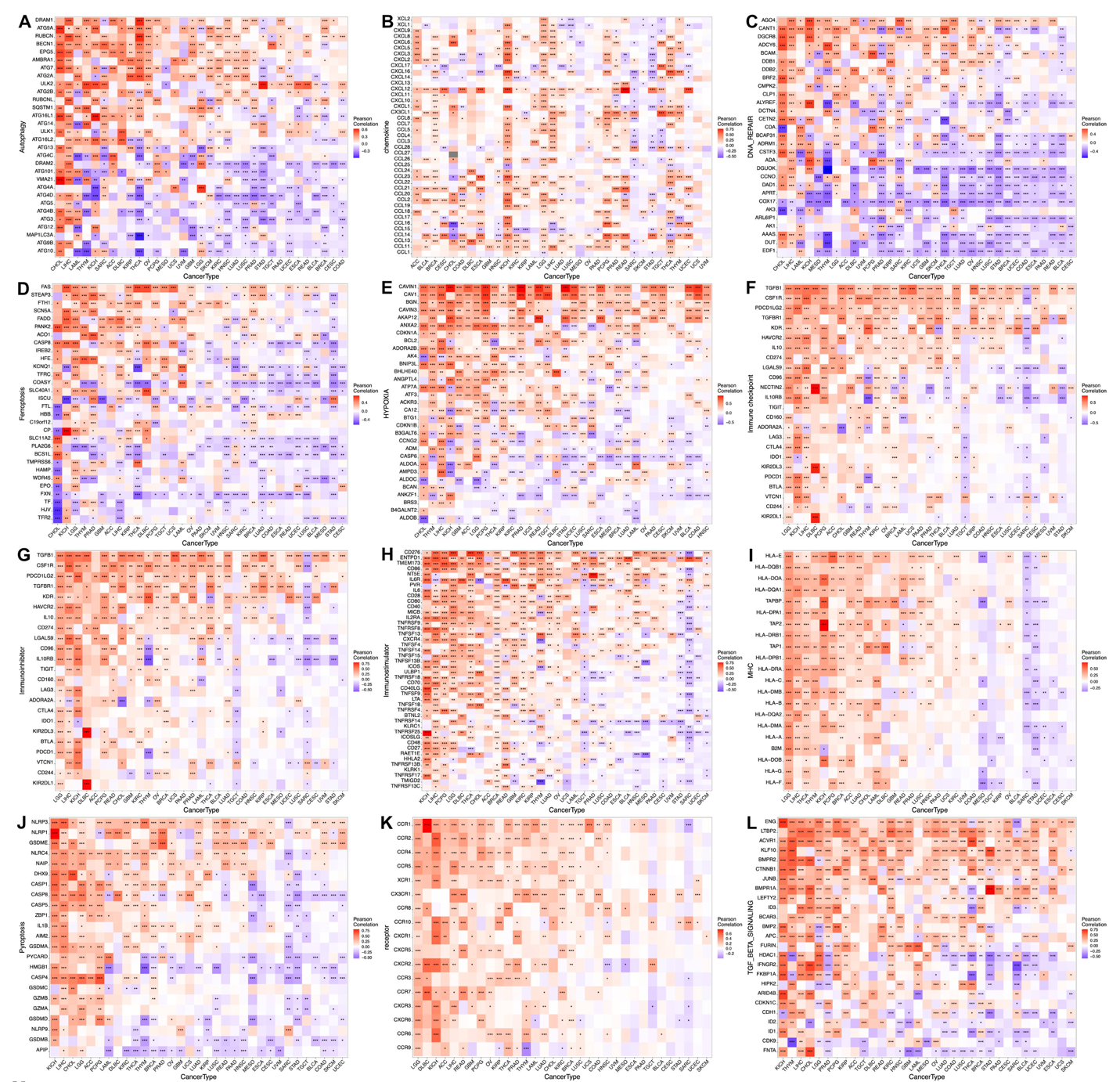


Figure S11. Correlation analyses between the expression of FLNB and crucial tumor regulatory pathways.


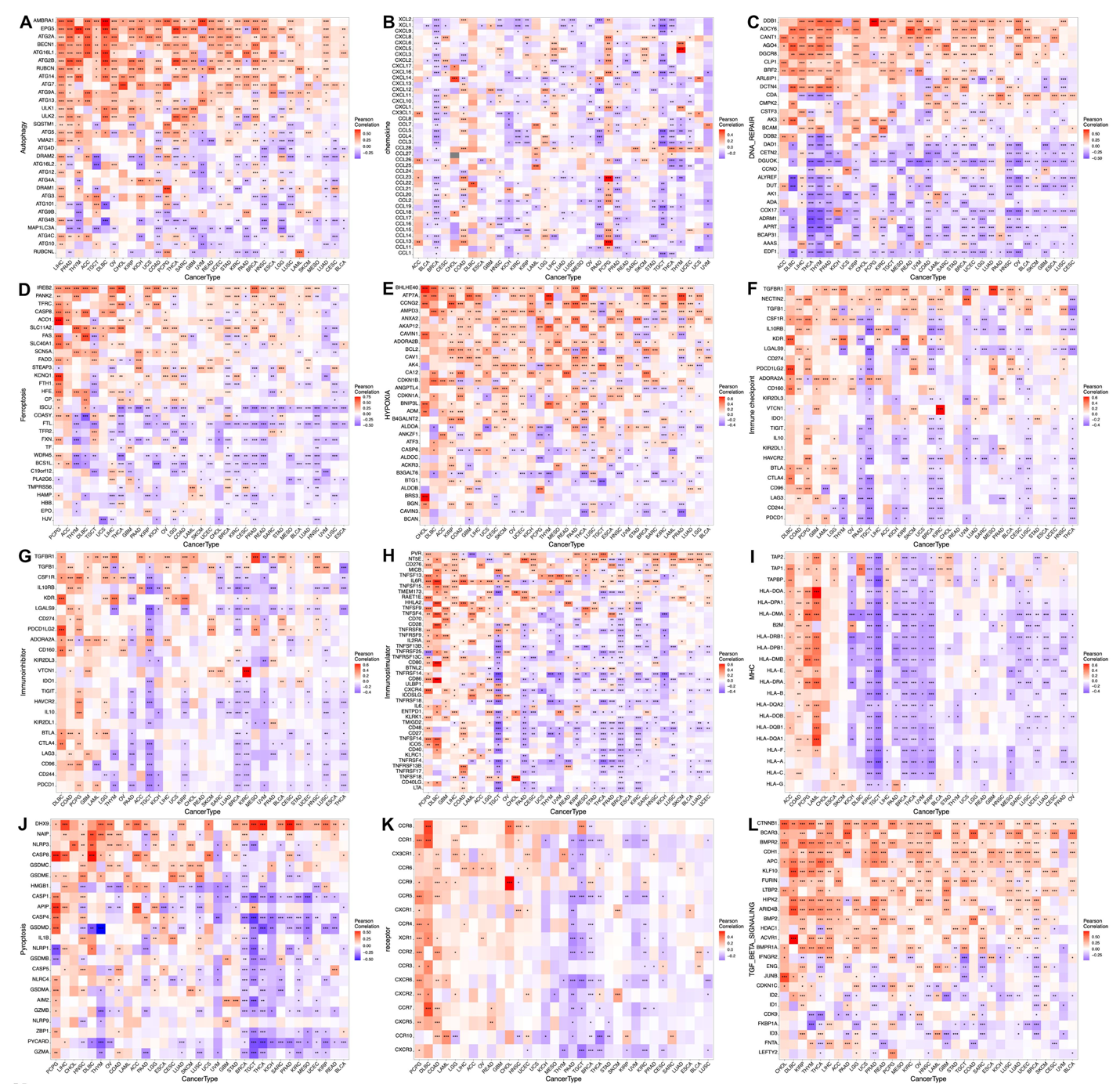


Figure S12. The expression of hub genes exhibited significant associations with tumor mutational burden (TMB), microsatellite instability (MSI), and neoantigen load (NEO).


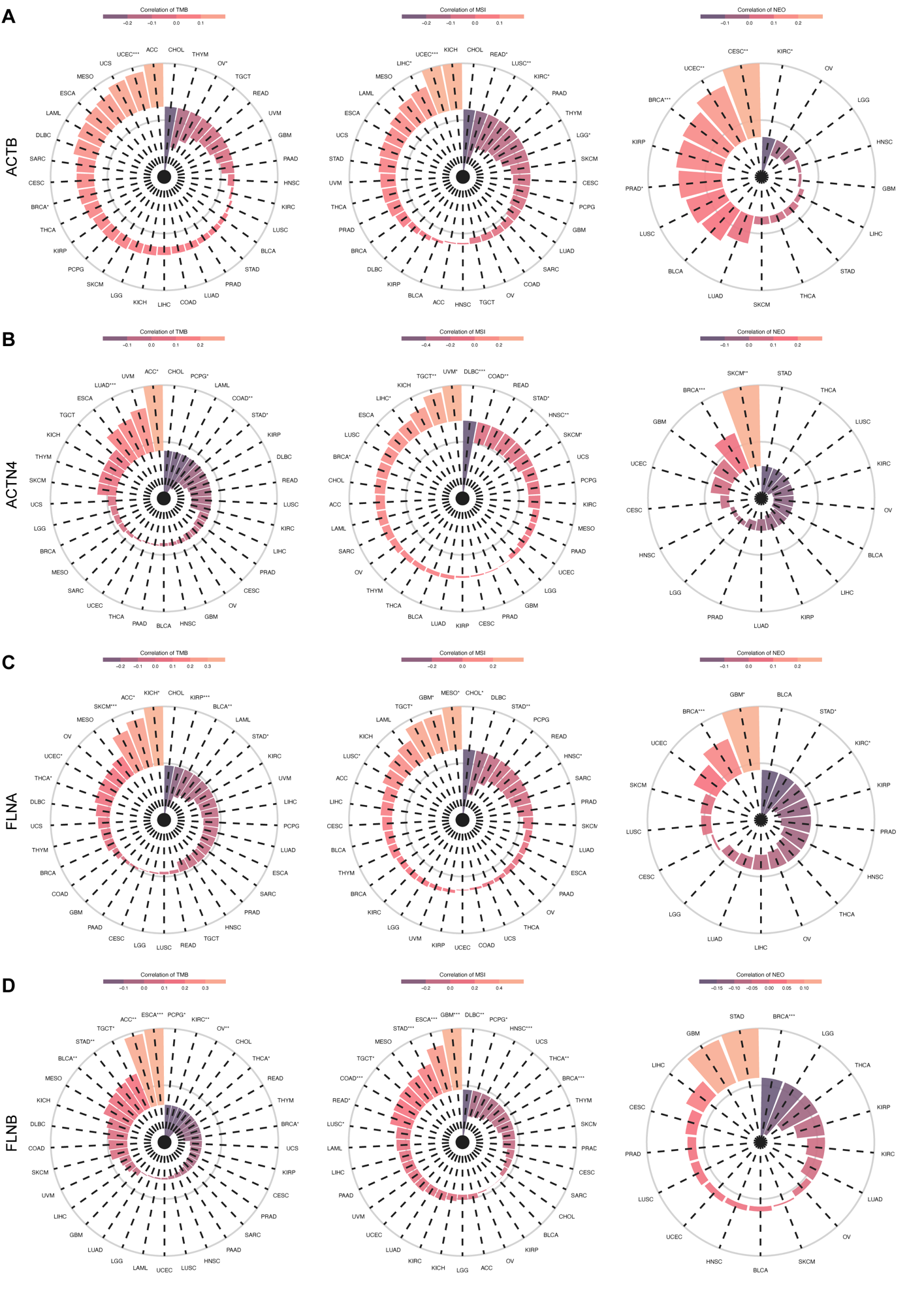


Figure S13. The role of DRGs in predicting the therapeutic efficacy of immunotherapy.


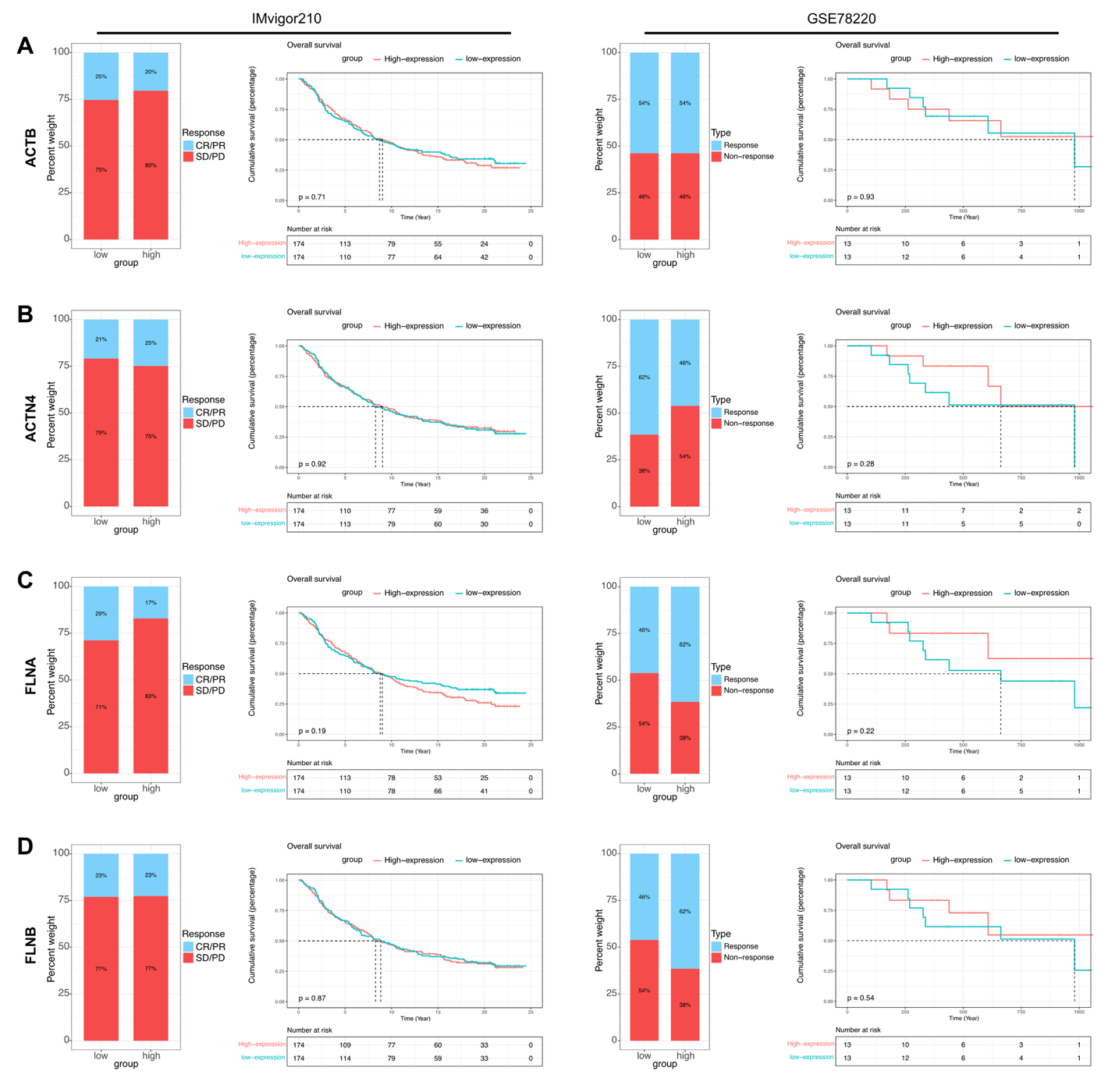


Figure S14. Mutational analysis of DRGs between high- and low-expression group.


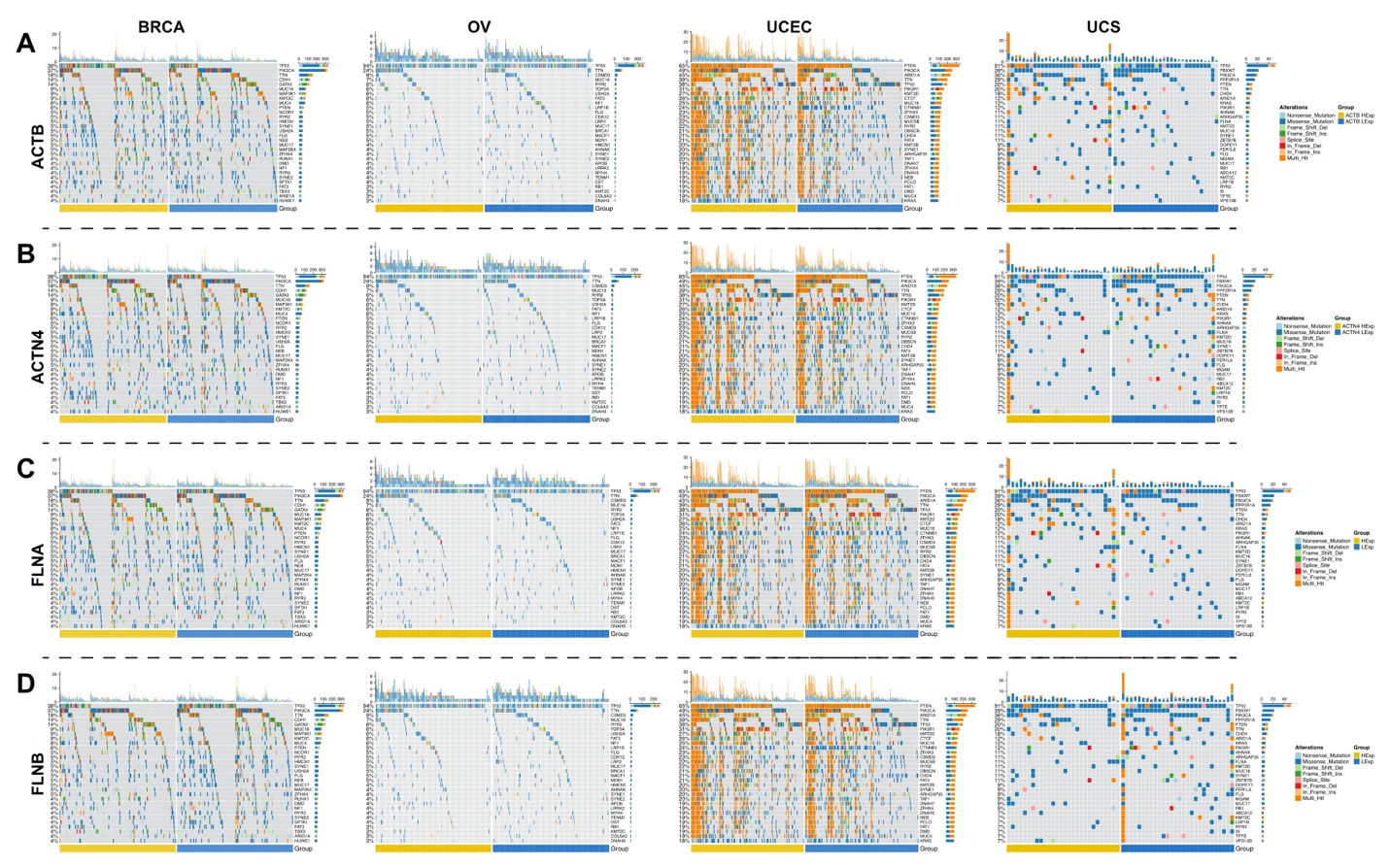


Table S1. Functional Annotation and Pathway Analysis of SLC7A11 and INF2 Genes Using Metascape.

Table S2. Details of Training and Test Set Samples for Specific Tumors in TCGA.

Table S3. Disulfidptosis-related genes (DRGs).

Table S4. The sequences of qPCR primers.

**Supplementary Methods**

LASSO model implementation

The glmnet package in R was used to perform LASSO Cox regression modeling. The 10-fold cross-validation was configured using cv.glmnet() with the argument nfolds = 10. The keep = TRUE option was used to retaian fold-specific predictions, and the random number generator seed was fixed using set.seed(111) to ensure reproducibility across runs. The optimal λ value was selected using the one-standard-error rule, balancing model complexity and predictive performance.
